# Economic factors promoting vaccine nationalism in the face of viral evolution

**DOI:** 10.1371/journal.pcbi.1014466

**Published:** 2026-08-31

**Authors:** Ari S. Freedman, Bjarke Frost Nielsen, Chadi M. Saad-Roy, Bryan T. Grenfell, C. Jessica E. Metcalf, Simon A. Levin

**Affiliations:** 1 Department of Ecology and Evolutionary Biology, Princeton University, Princeton, New Jersey, United States of America; 2 Department of Plant Biology, University of Vermont, Burlington, Vermont, United States of America; 3 Vermont Complex Systems Institute, University of Vermont, Burlington, Vermont, United States of America; 4 High Meadows Environmental Institute, Princeton University, Princeton, New Jersey, United States of America; 5 Niels Bohr Institute, University of Copenhagen, Copenhagen, Denmark; 6 PandemiX Center, Roskilde University, Roskilde, Denmark; 7 Miller Institute for Basic Research in Science, University of California, Berkeley, California, United States of America; 8 Department of Integrative Biology, University of California, Berkeley, California, United States of America; 9 Department of Mathematics, The University of British Columbia, Vancouver, Canada; 10 Department of Department of Microbiology and Immunology, The University of British Columbia, Vancouver, Canada; 11 Biodiversity Research Centre, University of British Columbia, Vancouver, Canada; University of Canterbury, NEW ZEALAND

## Abstract

The increasing interconnectedness of the modern world calls for globally equitable solutions to combat pandemic challenges. However, we have seen a tendency in recent decades for high-income countries to resort to “vaccine nationalism,” hoarding vaccine production to the detriment of lower-income countries. In addition, vaccine nationalism can prove detrimental to hoarding countries in the long term, as inequitable global vaccine distribution during the COVID-19 risked exacerbating the rise of harmful immune-escape variants that largely counteracted the original benefits of vaccine hoarding. Thus, vaccine hoarding may create a problem of time preference for a vaccine-producing country, where countries heavily discounting the future would opt for vaccine hoarding while countries lightly discounting the future would opt for vaccine sharing. Using a novel modeling framework integrating epidemiological, evolutionary, and economic processes, we demonstrate how high temporal discounting, low levels of outgroup prosociality, and high vaccine-distribution costs for low-income countries can promote vaccine-hoarding tendencies. We further show how these factors interact with epidemiological and evolutionary parameters to incentivize vaccine sharing in different ways: in some parameter regimes, vaccine sharing helps by reducing variant infections, while in others, vaccine sharing helps by reducing the probability of initial variant emergence. As a result, the optimal fraction of vaccines a country should share in our model is a bimodal function of the pathogen’s transmissibility. We thus provide a nuanced, model-based exploration of how various factors may contribute to vaccine nationalism’s emergence, emphasizing the need for international organizations to coordinate global vaccination responses to future pandemics.

## Author summary

In the wake of the extreme inequities in global vaccine distribution that arose during the COVID-19 pandemic, it is necessary to analyze the factors that drove countries to “vaccine nationalism” to ensure more equitable vaccine distribution for future pandemic challenges. Through a novel modeling framework combining epidemiological, evolutionary, and economic processes, we identify crucial economic and sociological factors that can promote vaccine-hoarding tendencies in high-income countries, despite the threat of viral evolution which could reverse the short-term benefits of vaccine hoarding. In particular, we highlight that shortsighted policies neglecting the roles of viral evolution and transmission in other countries promote vaccine hoarding, to the eventual detriment of the vaccine-hoarding country. To minimize global disease burden and probability of viral immune escape, we emphasize the need for international collaboration in overseeing global vaccine distribution, with policies that incorporate lower temporal discount rates, more prosocial thinking, and aid to vaccine-distribution infrastructure in lower-income countries.

## 1 Introduction

The phrase “disease knows no borders” is becoming increasingly relevant in our highly interconnected world [1]. As emerging infectious diseases have shown alarming capacity for rapid geographic spread in recent decades, it is prudent to treat pandemics as international challenges that require globally united solutions. However, pandemics often foster nationalistic tendencies as high-income countries scramble to protect their own citizens, even if the resulting detrimental effects to other countries may traverse borders to eventually offset the immediate benefits of nationalist policies [2].

This phenomenon is exemplified by “vaccine nationalism,” a practice vaccine-producing countries have employed across various pandemic threats (notably the 2009 H1N1 and 2020 COVID-19 pandemics) by hoarding vaccine stockpiles for their own citizens [3]. After the first two years of the COVID-19 pandemic, high-income countries still had over ten times as many vaccinations per capita as low-income countries [4]. As a result, it is widely speculated that the unequal distribution of vaccines significantly increased the global infection burden, driving the proliferation of SARS-CoV-2 immune-escape variants as countries with limited vaccine access produced larger outbreaks that were more likely to produce novel variants capable of immune escape [5–12]. Thus, vaccine nationalism likely acted against the best interests of vaccine-hoarding countries in the long-term by promoting the evolution of variants which have caused millions of deaths worldwide. Vaccine nationalism remains a relevant issue as the highly pathogenic avian influenza (HPAI) panzootic has shown worrying potential to cross over to humans [13], while the United States’ recent cuts at the time of this writing to international humanitarian organizations like the Global Alliance for Vaccines and Immunization (GAVI) [14]—which have lead previous pandemic vaccine-sharing efforts—may hinder our ability to control HPAI’s evolutionary potential or future spread in humans [15].

While previous studies have focused on the negative impacts of inequities in global vaccine distribution [5,6,16–21], little work has been done to uncover the economic incentives driving vaccine-hoarding behaviors in producing countries [22–24], especially with regards to vaccination’s role in preventing the evolution of immune-escape variants. We highlight three such potential factors. (i) The time preference with which a producing country makes its policy decisions dictates whether it prioritizes reducing its infections in the near-term through vaccine hoarding, or prioritizes reducing its own infections in the long-term through vaccine-sharing to minimize variant infections and emergence probability in all countries. This time preference can be formally represented by the country’s temporal discount rate [25]. Vaccine-producing countries operating under a high discount rate (whether imposed economically or arising psychologically from uncertainty [26,27]) may be disincentivized from vaccine sharing, as they will not consider future infections and the possibility of variant emergence very strongly in their decision-making calculus. (ii) Outgroup prosociality, or the degree to which a country considers another country’s welfare in addition to its own, may also be a contributing factor to a vaccine-producing country’s policy decisions, as vaccine sharing is effectively a prosocial act through the health benefits it provides to members of other countries [28]. (iii) Lastly, high costs for a lower-income country to develop the necessary infrastructure to distribute vaccines to its populace may deter a producing country from sending doses and risking the doses going unused. This was an especially prominent issue during the COVID-19 pandemic as the novel mRNA vaccines for SARS-CoV-2 required cold-chain storage that proved prohibitively expensive for many countries to implement [29], which may have contributed to vaccine-producing countries’ stockpiling tendencies.

In this work, we construct a novel two-country modeling framework capable of assessing the roles that these three economic factors—temporal discounting, prosociality, and development costs for vaccine-distribution infrastructure—play in shaping vaccine nationalism during a viral pandemic with the threat of escape variant emergence. Specifically, we find that high discount rates, low levels of prosociality, and high infrastructure development costs in receiving countries all contribute to the rise of vaccine nationalism across a broad range of parameters. Our framework further reveals how different vaccine-sharing policies and parameter regimes affect both epidemiological (amount of variant infections given emergence) and evolutionary outcomes (probability of variant emergence in the first place). Notably, we find that the optimal fraction of vaccines the producing country will share has a bimodal response to the virus’s basic reproduction number *R*_0_, with vaccine sharing having primarily epidemiological benefits when *R*_0_ is high and evolutionary benefits when *R*_0_ is low. Regarding vaccine-distribution infrastructure (e.g., cold-chain storage for novel mRNA vaccines) in the receiving country, we provide a mathematical criterion to determine how high infrastructure costs can be before forcing an equilibrium of suboptimal vaccine hoarding by the producing country. Lastly, we discuss the economic pressures that prevent the producing country from helping with the burden of infrastructure development, and the consequent need for well-funded international organizations to coordinate vaccine-sharing efforts.

### 1.1 Modeling framework overview

**Fig 1:**
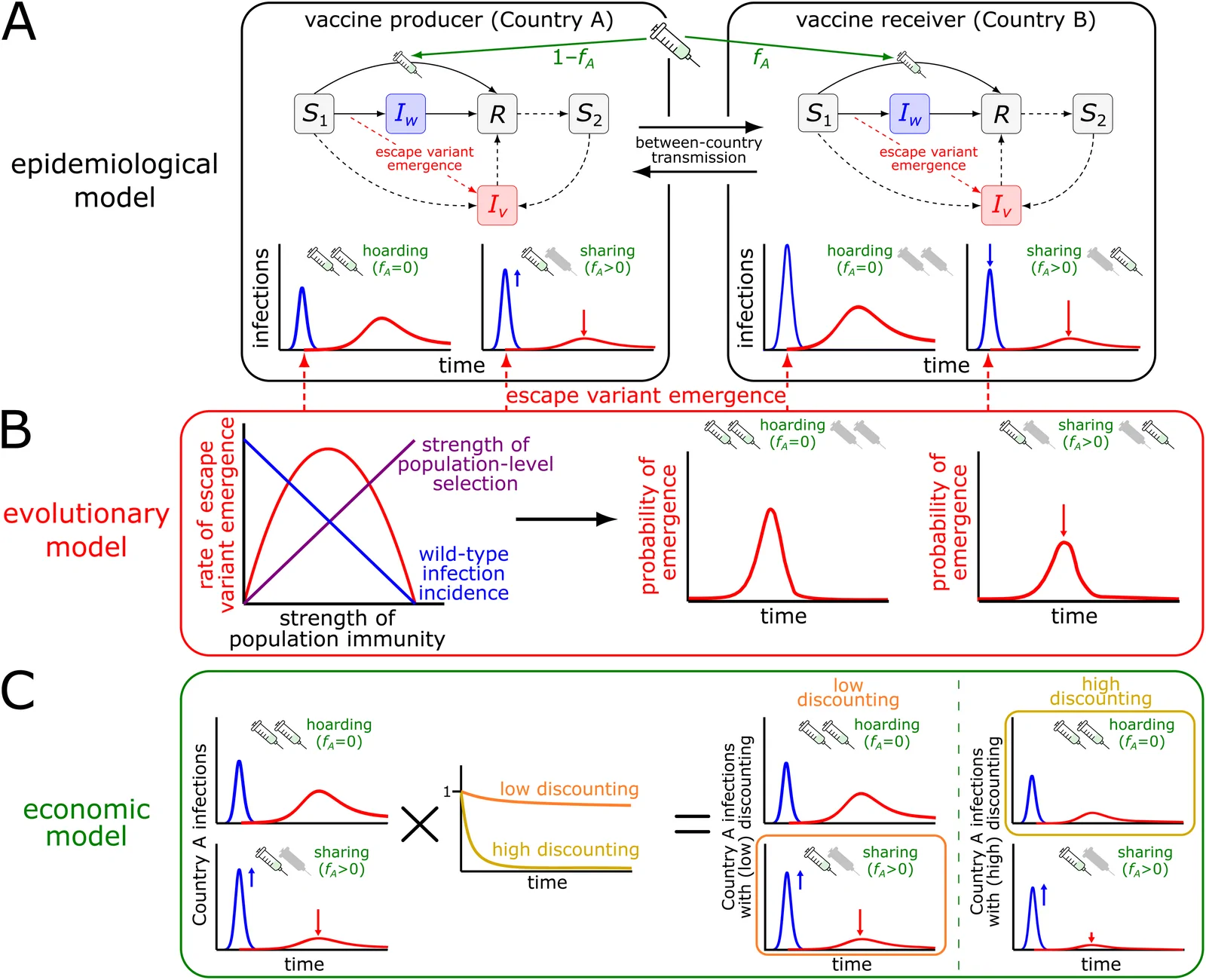
Overview of our modeling framework and motivation. **(A)** An epidemiological model of wild-type (blue) and immune-escape variant (red) strains of a respiratory virus spreading in two countries, coupled by between-country transmission and the vaccine-producing Country A sharing a fraction $f_{A}$ of its vaccines with the vaccine receiving Country **B.** Vaccine sharing increases Country A’s wild-type infections while reducing later infections from variants due to Country B’s reduced infections. **(B)** The evolutionary model extends the concepts of the population-scale phylodynamic curve (introduced by [30]) to predict the timing and probability of an escape variant emerging, which may be reduced by vaccine sharing. **(C)** The economic model defines an infection cost for Country A which discounts future infections and that Country A seeks to minimize through optimal vaccine allocation. Though vaccine sharing is favored under low discounting, vaccine hoarding is preferred if future variant waves are highly discounted. We also explore how prosociality and costs to vaccine distribution in Country B shape both countries’ vaccine allocation decisions in a game-theoretic framework. Small blue or red arrows show the projected impacts of vaccine sharing on wild-type or variant infections, respectively. More details in S1 Appendix.

Our modeling framework consists of a vaccine-producing “Country A” choosing what fraction $f_{A}$ of its vaccine production to share with a vaccine receiving “Country B,” based on the dynamics of a two-country disease model coupled with viral evolution and an associated infection cost which Country A is trying to minimize. The framework thus connects an epidemiological model, an evolutionary model, and an economic model (Fig 1). In the epidemiological model, the two countries’ dynamics are coupled by vaccine sharing and a small degree of between-country transmission. The evolutionary model uses population-level phylodynamic principles [30,31] to calculate the probability that an immune-escape variant with a 10% transmission increase will emerge in each country at each time point, given each country’s prevalence of the wild-type strain and immune landscape at that time. Crucially, this framework uniquely allows us to mechanistically calculate variant emergence probabilities at all points in continuous time while simultaneously tracking all of the different epidemic trajectories that would arise from variant emergence at every point in time.

The economic model then describes how each country calculates the total infection costs that result from a policy as its cumulative (wild-type and variant) incidence over time, discounted at an annual rate δ. In order to account for all the different possible times at which an escape variant emerges, their cost functions sum variant infections across all scenarios depending on variant emergence times, weighted by the probabilities of emergence at each time. Prosociality acts on a country’s infection cost by additionally incorporating a fraction ρ of the other country’s infections into the cost. And in later sections, we allow Country B agency in deciding how much it will invest in a costly new vaccine-distribution infrastructure, quantified by a cap $f_{B}$ on the share of vaccines it is able to accept. Consequently, Country A shares a fraction $f_{A}$ of its vaccines, but Country B ends up distributing only a fraction $\min\{f_{A},f_{B}\}$ to its populace, which may or may not be less than $f_{A}$ depending on the values of $f_{A}$ and $f_{B}$ that the two countries choose. We then explore how the high vaccine-distribution cost function $c(f_{B})$ to Country B must be to force a Nash equilibrium vaccine-sharing strategy in which Country A donates fewer vaccines than its preference, in order to avoid doses being wasted on an underfunded distribution infrastructure. We thus assume that, while vaccine distribution may be costly for the lower-income Country B, these costs are negligible to the higher-income Country A.

## 2 Results and discussion

### 2.1 Temporal discounting and prosociality

**Fig 2:**
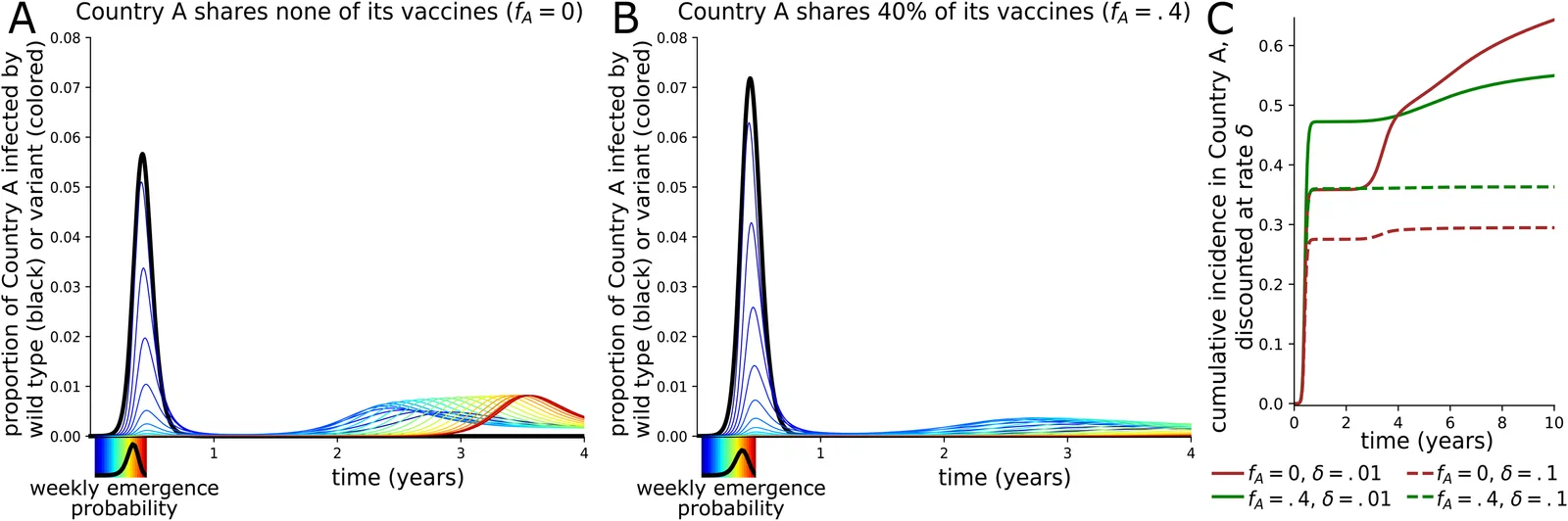
Example model output. **(A)–(B)** Wild-type (black) and variant (colors) prevalence in Country A, which either (A) hoards all vaccines or (B) shares 40% of vaccines with Country B. Each variant prevalence curve represents one of many parallel scenarios, stratified and colored by the week of variant emergence. Color bars map each color to its corresponding variant emergence week on the *x*-axis above it, with the curves inside showing the probability of a variant emerging in each week. **(C)** Country A’s cumulative infection cost, with infections discounted at annual rate δ of 1% (solid) or 10% (dashed), and with Country A either hoarding all vaccines (brown) or sharing 40% of its vaccines (green). The infection cost accounts for both wild-type infections and a weighted average of variant infections across all possible variant emergence scenarios. In this figure, to better demonstrate the model’s motivation, population-scaled daily mutation rate μ=5 and between-country coupling η=.1, while the default in other figures is μ=2 and η=.05. And for this and all other figures unless otherwise noted: wild-type *R*_0_ = 2, annual per-capita vaccine production rate ν=1, and the escape variant has a 10% transmission increase.

Fig 2 shows a typical model output for a viral infection with a wild-type basic reproduction number *R*_0_ of 2 (roughly the infectiousness of the original SARS-CoV-2 strains [32]), exemplifying the competing pressures that arise for a vaccine-producing country. Comparing Fig 2A, where Country A hoards all of its vaccines, to Fig 2B, where the vaccine-producing Country A shares 40% of its vaccines with the receiving Country B, we see that vaccine sharing produces a short-term setback for Country A with increased wild-type infections (black curve) but a long-term benefit with reduced later waves of variant infections (colored curves, each one representing a different scenario of variant emergence with a different emergence week). Fig 2C then shows the accumulated infection costs over time for Country A either sharing (red) or hoarding (green) vaccines and at an annual discount rate of 1% (solid) or 10% (dashed), with variant infections averaged over all possible variant trajectories weighted by their respective emerge probabilities (black curves inside color bars).

This example illustrates the intertemporal conflict that a vaccine-producing country faces and the importance of the discount rate. For both the low annual discount rate of 1% and the high discount rate of 10%, vaccine sharing is more costly in the first two years as having fewer vaccines exacerbates Country A’s initial wave of infections. These initial infections are mostly due to the wild-type virus, but early variant emergence is also possible albeit with very low probability (dark blue curves in Fig 2). However, vaccine sharing greatly reduces costs from variant infections in later years once the population’s initial wild-type immunity has waned (Fig D in S1 Appendix), ultimately resulting in vaccine hoarding being the costlier option if discounting is low, while high discounting effectively ignores the later benefits of vaccine sharing and maintains hoarding as the less costly option.

**Fig 3:**
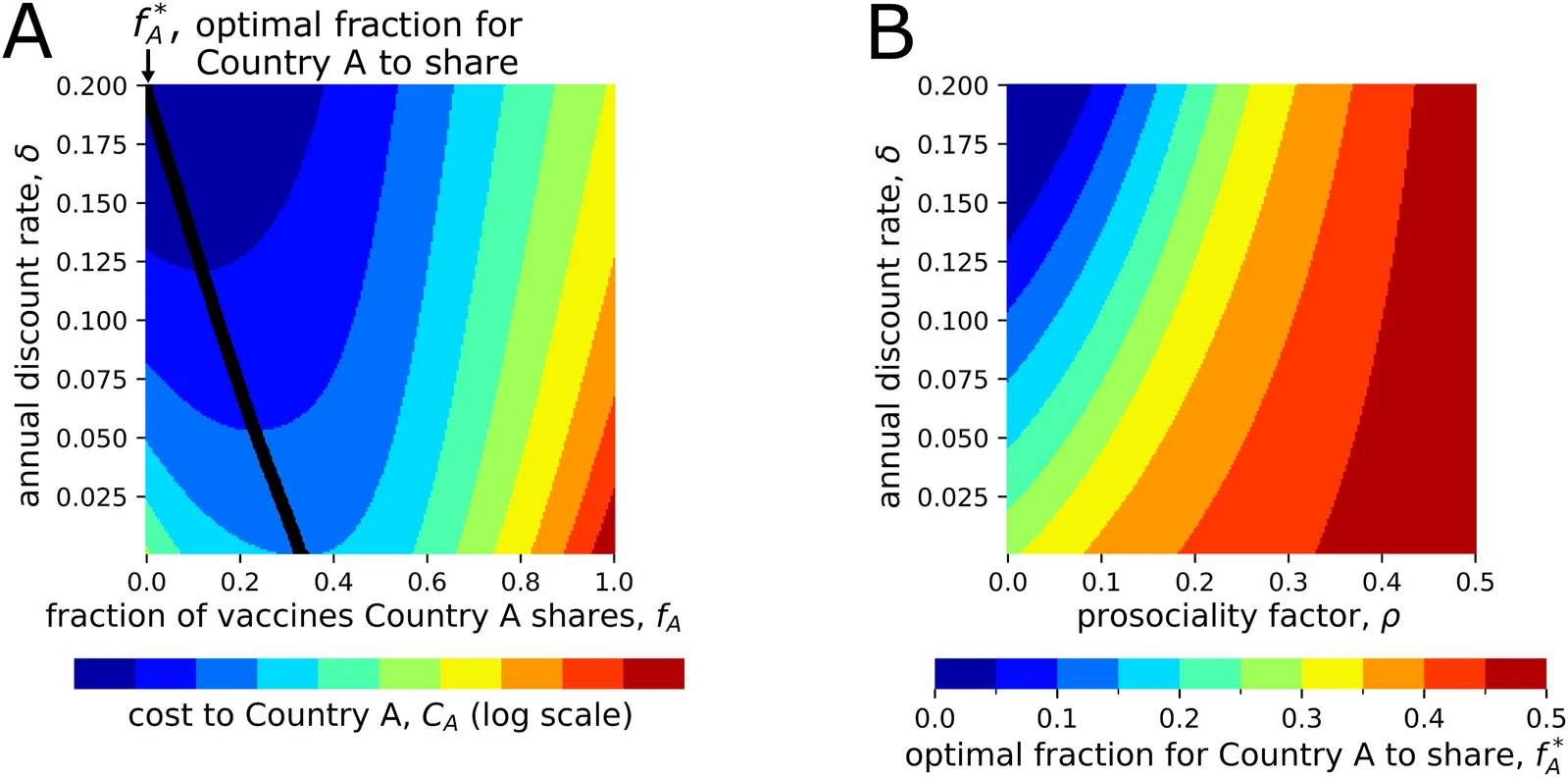
Effects of discounting and prosociality on optimal vaccine sharing. **(A)** Heatmap shows how the cumulative infection cost to the vaccine-producing Country A varies across the whole range of vaccine-sharing possibilities on the *x*-axis and with the *y*-axis varying the infection cost’s annual discount rate δ. The black curve shows the fraction $f_{A}^{*}$ of vaccines Country A shares that minimizes its infection cost $C_{A}$. **(B)** The optimal vaccine-sharing fraction $f_{A}^{*}$ for simultaneously varying discount rate δ and prosociality factor ρ (the fraction of Country B’s infection cost that Country A considers).

**Fig 4:**
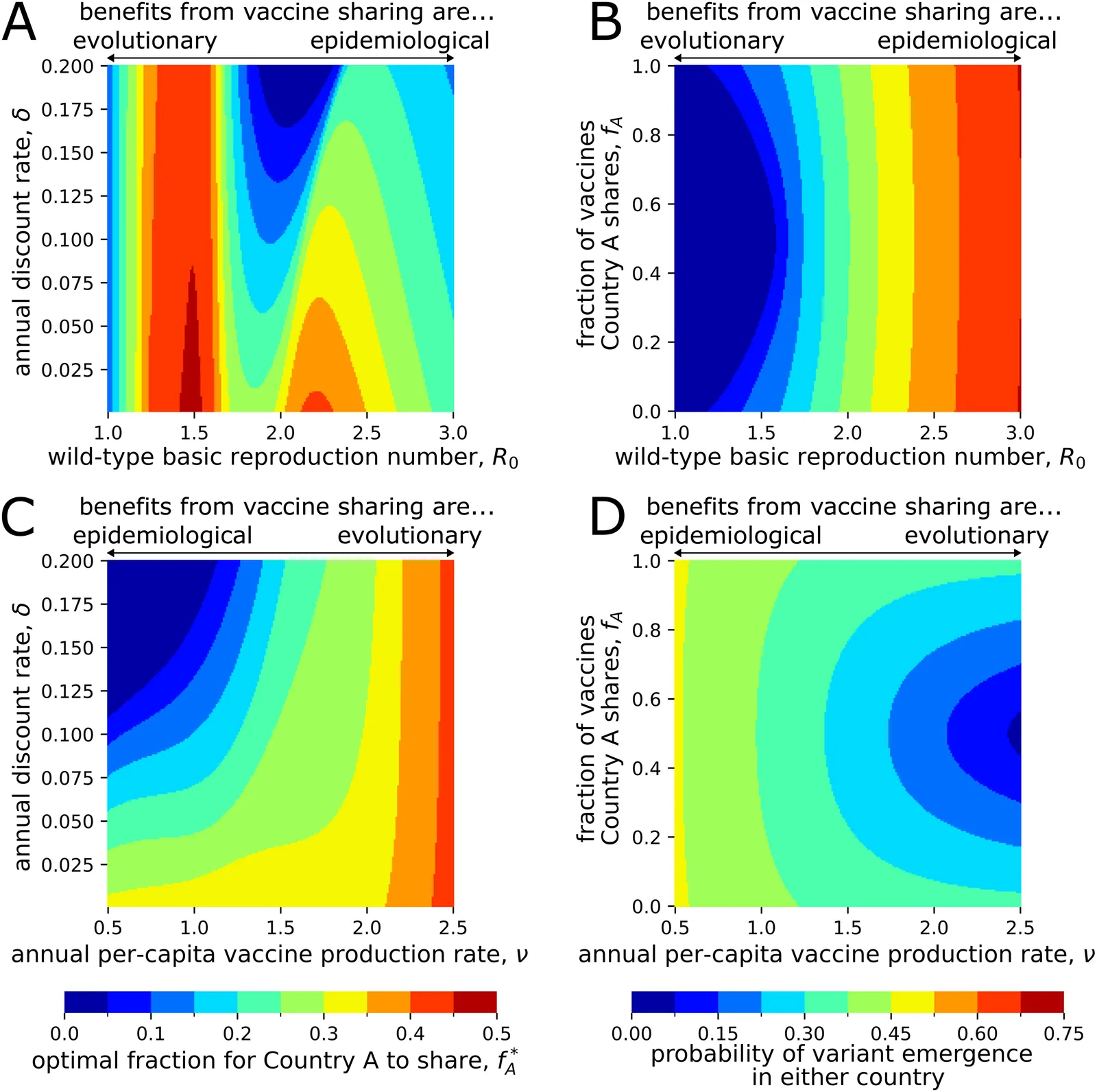
Effects of varying the wild-type basic reproduction number *R*_0_ (top row) or the annual per-capita vaccine production rate ν (bottom row), on the cost-minimizing fraction $f_{A}^{*}$ of vaccines for Country A to share (left column), and the probability of an immune-escape variant emerging in either country over the course of a simulation (right column). (A) and (C) also vary the infection cost’s annual discount rate δ; (B) and (D) also vary the fraction $f_{A}$ of vaccines Country A shares. In (B) and **(D)**, the variant *R*_0_ scales with the wild-type *R*_0_ so that the variant always has a 10% increase in transmissibility against primary susceptibles, while the variant’s reproduction number against secondary susceptibles is scaled down to 1 to standardize the model’s long-term behavior. Benefits from vaccine sharing are either primarily evolutionary (reducing variant emergence probability) when wild-type *R*_0_ is low or vaccine production is high, or primarily epidemiological (reducing variant cases given emergence) when wild-type *R*_0_ is high or vaccine production is low.

**Fig 5:**
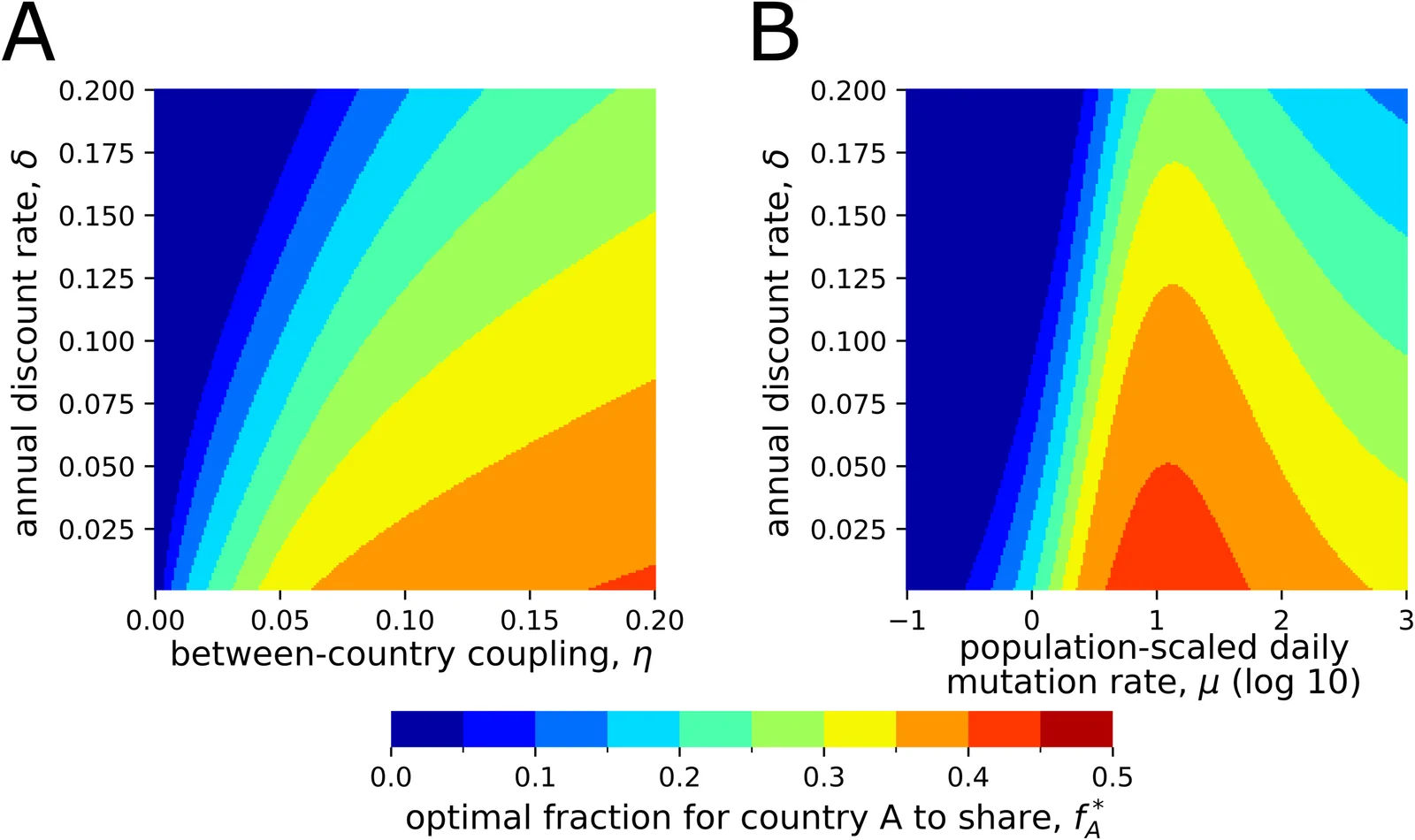
Effects of varying the virus’s population-scaled daily mutation rate μ. (A) or the degree of coupling between the two countries $\begin{array}{c} \eta \end{array}$ (B, measured as the fraction of interactions that occur between people of different countries) on the cost-minimizing fraction $\begin{array}{c} f_{A}^{*} \end{array}$ of vaccines for Country A to share. Both (A) and (B) also vary the infection cost’s annual discount rate $\begin{array}{c} \delta \end{array}$.

The pattern that high temporal discounting favors vaccine hoarding appears universally from this model. Fig 3A, which visualizes the infection cost to Country A across all possible vaccine-sharing options and for a range of annual discount rates, shows that there is a clear single cost-minimizing vaccine-sharing fraction $f_{A}^{*}$ (black curve) which decreases with higher discount rates. The optimal $f_{A}^{*}$ decreasing with higher discount rates remains true for a wide variety of other parameter regimes, as heatmaps plotting $f_{A}^{*}$ for discount rates varying along with a host of other parameters show (Figs 3B, 4A, 4C, and 5). The impact of varying the discount rate is quite significant on the optimal vaccine-sharing fraction: in the absence of any prosociality, Country A can go from optimally hoarding all vaccines ($f_{A}^{*}=0$) at 20% annual discounting to optimally sharing about 30% of vaccines ($f_{A}^{*}\approx \mathrm{.3}$) at 1% annual discounting. Fig 3B also shows this effect of discounting on $f_{A}^{*}$ in conjunction with the effect of prosociality. We incorporate prosocial behavior by having Country A’s cost function include a fraction ρ of the infection cost from Country B. As expected, increasing this prosociality factor boosts the proportion of vaccines Country A will share. Mechanistically, prosociality has this effect because hoarding vaccines universally hurts Country B in both wild-type and variant infections (Fig D in S1 Appendix); thus, accounting for Country B’s welfare deters against vaccine hoarding.

Taken together, these results demonstrate that vaccine-hoarding tendencies can be promoted by temporal discounting and a lack of prosociality—in other words, by disregarding infections (and viral evolution) in the future and in other countries. Times of crisis and increased nationalism such as pandemics may trigger greater temporal discounting in decision-making and declines in outgroup prosociality, as it seemingly becomes more important to invest resources into managing the crisis at the present time and place [2,22]. Uncertainty in the future can also discourage consideration of future outcomes [26,27,33]; this can be seen from the COVID-19 pandemic as the potential for destructive variant evolution was widely underestimated in the pandemic’s early stages [34]. Thus, high temporal discounting and low levels of international prosociality are likely contributors to vaccine nationalism during pandemics, specifically in the cases of the 2009 H1N1 and COVID-19 pandemics. Furthermore, these two factors may be closely linked, as experiments have uncovered correlations between individuals’ propensities for discounting future outcomes and eschewing prosocial behavior [35].

It is worth noting that the discount rate we model may in practice be a combination of government-appointed economic discount rates as well as psychologically-induced time preferences, which are known to increase with level of uncertainty in future outcomes [33,36]. Similarly with prosociality, our model greatly simplifies how prosociality functions, making no distinction between policy-mandated and psychologically-based prosociality in their influences on policy decisions. Also, people typically discount the future “hyperbolically,” discounting the far future similarly to the near future [37,38], but we do not explore that possibility here.

### 2.2 Vaccine sharing’s epidemiological and evolutionary benefits

As we have seen, vaccine sharing provides long-term benefits in the form of reduced future costs from escape variant infections, and these benefits can outweigh the extra wild-type infections that reduced vaccination causes in the short-term if temporal discounting is sufficiently low. But what are the mechanisms by which vaccine sharing reduces variant infections?

Costs due to variant infections can be reduced in two ways. Since we model infection costs due to variants as the sum of all possible variant trajectories weighted by the probabilities that each trajectory will occur (based on their emergence time and our evolutionary model), variant costs can be reduced by either controlling variant infections given emergence—an “epidemiological benefit”—or by controlling the probability of variant emergence in the first place—an “evolutionary benefit.” In general, we see that vaccine sharing consistently lowers the overall probability of variant emergence—despite increasing emergence probability in Country A (Fig G in S1 Appendix)—when Country A shares fewer than half of its vaccines. However, the vaccine sharing’s effectiveness in lowering emergence probability and providing an evolutionary benefit is largely dependent on other parameters. We examine how the wild-type’s basic reproduction number and the total vaccine production rate impact whether vaccine sharing’s benefits are primarily epidemiological or evolutionary.

For a wild-type basic reproduction number of *R*_0_ = 2, 40% vaccine sharing between countries ($f_{A}=\mathrm{.4}$) as compared to complete vaccine hoarding ($f_{A}=0$) only reduces the overall probability of variant emergence from 40% to 37% (Fig 4B, or areas under the emergence probability curves in Fig 2A and 2B). The benefit of vaccine sharing is thus mainly epidemiological rather than evolutionary, drawing from the substantial decrease in variant infections given variant emergence (as seen in Fig 2A and 2B). This decrease in variant infections given emergence is a product of the reduced population-level susceptibility in Country B that access to vaccination provides them; however, this decrease in susceptibility does not happen soon enough to have a large effect on dampening the probability of variant emergence (Fig D in S1 Appendix).

By contrast, with a lower wild-type *R*_0_ of 1.5, equitable sharing results in a more significant decrease in emergence probability from 18% to 2% (Fig 4B), while Country A’s variant infections given emergence are not substantially reduced (Figs E and F in S1 Appendix). Thus, the benefit from vaccine-sharing is primarily evolutionary when wild-type *R*_0_ is low. The difference lies in the fact that when the basic reproduction number is sufficiently low, vaccine sharing is able to lower the reproduction number in both countries below 1, curbing the outbreaks and evolutionary potential in both countries. So when wild-type *R*_0_ is low, vaccine sharing actually reduces Country A’s wild-type infections (contrary to when wild-type *R*_0_ is higher as in Fig 2), though the cost this saves is small compared to the cost reduction resulting from the lower emergence probability (Fig E3 in S1 Appendix).

When wild-type *R*_0_ is low, the benefit from vaccine sharing also occurs much earlier. Consequently, the temporal discount rate has little effect on Country A’s optimal vaccine-sharing strategy for small wild-type *R*_0_ (Fig 4A). Raising the vaccine production rate ν has a similar effect as lowering wild-type *R*_0_, in that both render vaccine sharing more effective at rapidly controlling the pandemic before variant emergence can occur. We thus see that higher vaccine production rates also result in vaccine sharing having a greater evolutionary benefit (Fig 4D) and discount rate having a lesser impact on Country A’s preferred vaccine-sharing strategy (Fig 4C).

Unless otherwise noted, we focus on the case of higher wild-type *R*_0_ = 2 and lower ν=1, corresponding to the regime where vaccine sharing has a primarily epidemiological benefit, as this is the more likely case for an emerging epidemic and the more interesting one to study with regards to temporal discounting. However, we more thoroughly examine the case of the lower wild-type *R*_0_ = 1.5 with Figs E and F in S1 Appendix.

### 2.3 Impacts of epidemiological and evolutionary parameters on vaccine sharing

The optimal vaccine-sharing strategy for the producing country responds in interesting ways to some of the model’s key epidemiological and evolutionary parameters, yielding important insights into the biological factors influencing vaccine nationalism. Most interestingly, the optimal fraction $f_{A}^{*}$ that Country A shares is a bimodal function of the wild-type basic reproduction number *R*_0_ (Fig 4A), each mode corresponding to one of the two regimes discussed in the previous section for how vaccine sharing is beneficial. The first peak for lower wild-type *R*_0_ of 1.2–1.7 corresponds to the “evolutionary benefit” regime, where vaccine sharing’s greatest impact is in preventing the early rise of escape variants by bringing both countries’ effective reproductive numbers below 1; and the second peak for higher wild-type *R*_0_ of 2–3 corresponds to the “epidemiological benefit” regime, where vaccine sharing’s impact occurs later on in reducing variant infections given emergence without appreciably changing emergence probability. In between these two regimes there is a drop in the effectiveness of vaccine sharing when wild-type *R*_0_ is around the 1.7–2 range. And on either extreme, for very high wild-type *R*_0_ or *R*_0_ very close to 1, vaccine sharing is also ineffective because, respectively, the virus is too strong to be controlled by the total available vaccine production or the virus is too weak to warrant vaccine-related expenditures.

The vaccine production rate ν similarly follows the general trend that the regime in which vaccine sharing provides evolutionary benefits (high ν) incentivizes greater vaccine sharing than the regime in which vaccine sharing provides epidemiological benefits (low ν). This pattern can be more simply understood by the fact that when Country A has more vaccine production, it becomes less costly to give some of that vaccine production away as it will still have enough vaccination to control its own infections. However, the response of the optimal vaccine-sharing fraction $f_{A}^{*}$ to ν is simply monotonic (unlike its bimodal response to wild-type *R*_0_), under this parameterization where variants have a 10% transmission advantage.

Vaccine sharing can still be an optimal strategy even when the vaccine has a 10% transmission decrease (as opposed to the 10% increase we explore in our main results), as we show with Fig I in S1 Appendix across a wide range of parameters. Thus, variant transmission increases (the status quo in vaccine nationalism models) are not necessary to incentivize vaccine sharing: we see that vaccine sharing can still be optimal to reduce the long-term threat of variant infections even when the variant is less transmissible than the wild-type, though the optimal vaccine-sharing fraction $f_{A}^{*}$ is generally lower in this case. Intriguingly, $f_{A}^{*}$ does have a bimodal response to vaccine production rate ν in this weaker-variant parameterization (Fig I3 in S1 Appendix).

The degree of between-country coupling η and the virus’s immune-escape mutation rate μ also factor into the vaccine producer’s optimal sharing strategy (Fig 5). The optimal $f_{A}^{*}$ monotonically increases with the strength of coupling, measured by the fraction of interactions a person has with another country, as the damages to Country B incurred by vaccine hoarding are more detrimental to Country A in the long-term when the two countries are tightly linked (Fig 5A). Similarly, higher mutation rates also exacerbate the danger from variant infections to further incentivize vaccine sharing, but only up to a point. Above a threshold (μ≈10–15), variant emergence becomes so likely that vaccine sharing loses value as a tool to control variant emergence, leading to a unimodal response of $f_{A}^{*}$ to mutation rate (Fig 5B). We also find that the pattern of vaccine sharing having a stronger evolutionary impact when wild-type *R*_0_ is low or vaccine production rate is high still holds true under a range of different mutation rates (Fig H in S1 Appendix).

### 2.4 High costs for vaccine-distribution infrastructure encourage vaccine hoarding

We now extend the economic model to incorporate costs to developing novel vaccine-distribution infrastructure for the receiving country, allowing it decision-making agency in choosing how much infrastructure it will invest in. Utilizing a game-theoretic framework, Country B now decides on a maximum fraction $f_{B}$ of the total vaccine production it is willing to accept, while Country A simultaneously and independently decides what fraction $f_{A}$ of vaccines it will share. Effectively, Country B vaccinates its populace at rate $\min\{f_{A},f_{B}\}\nu$, with $f_{B}$ acting as a cap to the amount of vaccines Country B can accept due to its limited vaccine-distribution infrastructure (while Country A still vaccinates itself at rate $(1-f_{A})\nu$). The up-front cost to developing vaccine-distribution infrastructure is added to Country B’s infection cost in the form of the vaccine-distribution cost function $c(f_{B})$.

**Fig 6:**
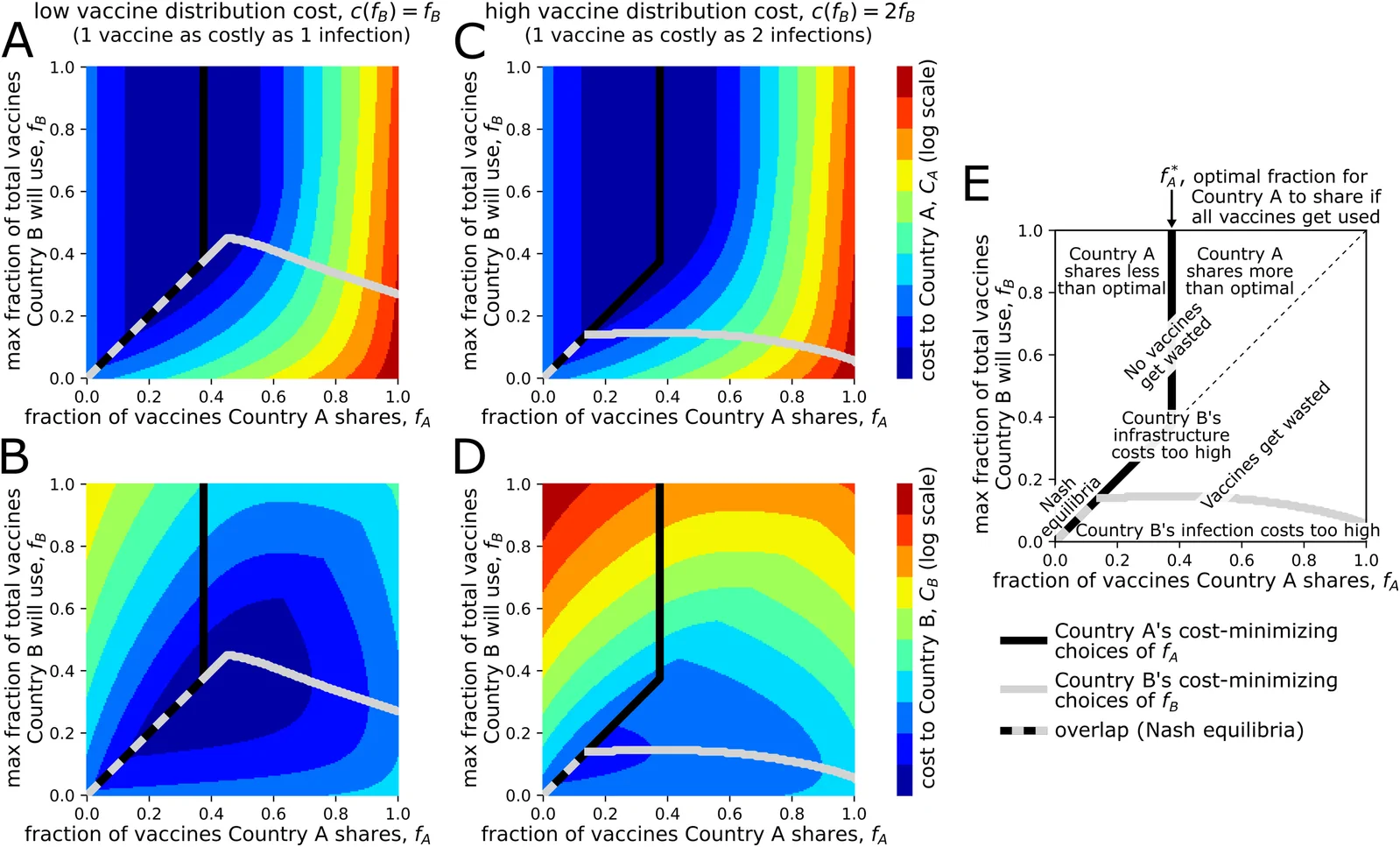
Infection costs to Country A ($C_{A}$, top row) and Country B ($C_{B}$, bottom row) with *x*-axes varying $f_{A}$ (fraction of its vaccines that Country A shares with Country B) and *y*-axes varying $f_{B}$ (the maximum fraction of vaccine production that Country B is able to accept), and with different vaccine-distribution costs to Country B. In the left column **(A,B)**, there is a low vaccine-distribution cost function $c(f_{B})=f_{B}$, where distributing one vaccine is as costly as one average infection; and in the middle column **(C,D)**, there is a high cost function $c(f_{B})=2f_{B}$, where one vaccine is as costly as two infections. Black curves show the values of $f_{A}$ that minimize Country A’s infection cost for each value of $f_{B}$, gray curves show the values of $f_{B}$ that minimize Country B’s infection cost for each value of $f_{A}$, and their overlaps (black-gray dashed) represent regions of Nash equilibria. **(E)** Qualitative outcomes for each country in $f_{A}-f_{B}$ space: Country A and Country B both balance between sharing and accepting, respectively, more or fewer vaccines than is optimal for them, with the possibility that some vaccines go to waste (when $f_{A}>f_{B}$). This figure uses population-scaled daily mutation rate μ=5 to better illustrate these concepts.

We analyze the joint decision-making processes of the two countries through heatmaps plotting each country’s total costs (infection costs for Country A and infection plus vaccine-distribution costs for Country B) for all possible values of $f_{A}$ and $f_{B}$, along with curves showing the cost-minimizing choice of $f_{A}$ for each value of $f_{B}$ (black) and the cost-minimizing choice of $f_{B}$ for each value of $f_{A}$ (gray; Fig 6). Wherever these two curves overlap represents a Nash equilibrium, meaning neither country would choose to change its strategy given the other country stays constant. Given perfect information about each others’ costs, the two countries should decide on a strategy set $\left(f_{A},f_{B}\right)$ that is a Nash equilibrium, at least one of which is guaranteed to exist [39].

No matter the cost function, all equilibria $\left(f_{A},f_{B}\right)$ occur on the line $f_{A}=f_{B}$, since Country B would prefer to not invest an unnecessarily large amount in vaccine infrastructure ($f_{B}>f_{A}$) and Country A would prefer not to have vaccines go to waste by sharing more than Country B can distribute ($f_{A}>f_{B}$). Furthermore, all equilibria occur for $f_{A}\leq f_{A}^{*}$ (with $f_{A}^{*}$ Country A’s optimal vaccine-sharing fraction assuming Country B distributes all vaccines), since Country A will not share more vaccines than is optimal for them even if Country B is able to accept more. When vaccine-distribution costs are on the lower end relative to infection costs ($c(f_{B})=f_{B}$, Fig 6A and 6B) or there are no vaccine-distribution costs at all ($c(f_{B})=0$, Fig J1 and J2 in S1 Appendix), there are equilibria for all possible $f_{A}\leq f_{A}^{*}$; in particular, Country A is able to safely share its optimal vaccine allocation $f_{A}^{*}$ without worries that any vaccines will be wasted on an underfunded vaccine-distribution infrastructure in Country B.

However, these worries become realized when Country B’s distribution costs are sufficiently high ($c(f_{B})=2f_{B}$, Fig 6C and 6D). In this case, Country B is still able to invest in some vaccine-distribution infrastructure, but only a very limited amount, as the benefits of accepting more vaccines become outweighed by the costs. At extreme distribution costs ($c(f_{B})=4f_{B}$, Fig J3 and J4 in S1 Appendix), it becomes infeasible for Country B to accept any vaccines at all and $\left(f_{A},f_{B})=(0,0\right)$ becomes the only equilibrium. In both instances, Country A is unable to share its preferred fraction $f_{A}^{*}$ due to Country B’s lack of distribution infrastructure. We note that both $c(f_{B})=f_{B}$ and $c(f_{B})=2f_{B}$ represent situations where infection cost is on a similar order of magnitude to vaccination costs, which may be true for a disease with a low infection fatality rate (such as COVID-19), but for a disease with much higher infection fatality rate it may represent a situation closer to $c(f_{B})=0$ as vaccination costs may be negligible relative to very high infection costs.

If we only consider linear distribution cost functions of the form $c(f_{B})=\alpha f_{B}$, with α representing the cost of distributing one vaccine relative to one infection, then we can predict the exact value of α for which Country B’s distribution costs become too high for Country A to share its optimal $f_{A}^{*}$ (Fig B in S1 Appendix). Specifically, we prove in S1 Appendix that Country A sharing its optimal $f_{A}^{*}$ is a Nash equilibrium if and only if α is less than Country B’s marginal increase in infection cost from declining one vaccine when $f_{A}^{*}$ is shared. In general, Country A will share its desired amount of vaccines only when the infection cost for Country B to accept them all outweighs the distribution cost. For the parameter regime we present here, this α≈1.25 (Fig B in S1 Appendix; it is apparent that this α must be between 1 and 2 from visually interpolating between the high and low distribution cost cases in Fig 6).

Thus, high costs to developing novel vaccine-distribution infrastructure in other countries can dissuade a vaccine-producing country from sharing its stock, to avoid doses going unused. SARS-CoV-2 vaccine wastage was in fact observed during the pandemic as some low-income countries were unable to fund the appropriate cold-chain storage facilities across their more rural areas, while vaccine access to other countries was thwarted by natural disasters [40]. And any uncertainty in other countries’ ability to successfully distribute vaccines would further dissuade producers from sharing doses, even to countries that may have the necessary infrastructure. As a result, economic hardships and uncertainties in low-income countries’ distribution infrastructures may contribute to stockpiling by vaccine-producing countries.

If we further modify the model to allow the producing country to help shoulder some of the lower-income country’s infrastructure costs, this may allow the lower-income country to invest more in infrastructure to distribute more vaccines, leading to a scenario better for both countries. However, such a strategy is infeasible for the producing country from a game-theoretic standpoint, as we discuss in more detail in Fig K in S1 Appendix. Faced with a decision to help pay some portion of Country B’s infrastructure development costs, Country A at equilibrium will deny helping with infrastructure development, since it would receive no direct benefit from doing so without Country B simultaneously accepting more vaccines. But the simultaneous combination of Country A helping with infrastructure costs and Country B accepting more vaccines can result in a more optimal strategy for both countries, revealing a discrepancy between the realized Nash equilibrium and what is actually optimal for both countries. This emphasizes the need for international organizations to act as central planners in problems of vaccine allocation and, especially, development of vaccine-distribution infrastructure. Indeed, much of the help with creating cold-chain infrastructure to distribute SARS-CoV-2 vaccines in low-income countries has come from international humanitarian organizations [41].

This work only considers two countries, one a vaccine producer and the other a vaccine receiver. Future work could consider more countries interacting in a network of vaccine production and sharing. Just as free-riding can emerge among a group of individuals deciding whether to vaccinate or take advantage of the population-level immunity that arises from others vaccinating [42,43], free-riding may emerge among a group of vaccine-producing countries deciding whether to share vaccines with lower-income countries or take advantage of the benefits from other producing countries sharing their vaccines. Exploring the conditions in multi-country systems for which free-riding emerges is a salient avenue for future research.

Future work may also benefit from drawing parallels to climate modeling literature focusing on how to foster multi-national collaboration to abate climate change. Just as mutual compliance with international climate accords will be crucial for effective climate change mitigation [44,45], we have shown how global vaccine distribution can act as a common pool resource to limit infectious disease spread and harmful variant evolution. A fundamental difference between pandemics and climate change, however, is that of time scale: pandemic scenarios unfold over the course of just several years, which may make mitigation more challenging due to the need for immediate action, but possibly easier to spur countries to action due to the highly visible and rapidly evolving nature of emerging pandemics.

## 3 Caveats

Our modeling framework is greatly simplified from reality across all three of its component models, in order to make the framework broadly generalizable to a range of possible pandemic scenarios. We briefly summarize the main simplifications here, highlighting potential future directions to address them.

The epidemiological model that forms the basis of our framework assumes mass-action transmission, a prevalent assumption across the infectious disease model literature but one that ignores important heterogeneities in peoples’ transmissibility, susceptibility, and contact structures [46–48]. The model also assumes the vaccine perfectly blocks transmission against the wild-type virus but allows transmission against all variants at the same reduced rate. All of the model’s parameters are constant throughout time, while in reality, transmission rates may vary with seasonality and adoption of non-pharmaceutical interventions, and vaccination rates may vary with changes to production or vaccine hesitancy [49,50]. The parameters are also all constant between the two countries (aside from the vaccination rates controlled by $f_{A}$ and $f_{B}$), though different population sizes, contact structures, and social norms can lead to very different transmission rates between countries [51]. And the range of reproduction numbers we do explore here (1–3 for wild-type, 10% increase or decrease for variants against primary susceptibles, and 1 for variants against secondary susceptibles) only represents a small portion of the possible reproduction numbers that have been observed in human viral infections [52].

The evolutionary model prescribing the timing and evolution of viral variants is also simplified and descriptive of just one particular evolutionary pathway. Namely, we assume all evolution happens at transmission (rather than during infection) and all infections are equally likely to lead to variant emergence, following [30] from which our evolutionary model is derived, although evidence from SARS-CoV-2 suggests that the majority of variant evolution happened in a small minority of prolonged infections in immunosuppresed hosts [53,54]. We also assume only one specific variant may arise and that immunity against it is imperfect, although this could also be interpreted as different but identical variants arising sequentially each with perfect self-immunity but imperfect cross-immunity. Either way, our model’s individuals regularly vaccinate throughout their lives to keep boosting their immunity against the variant(s), a practice which in reality is recommended only for a small group of viral infections.

Lastly, in the economic model we ignore individual decision-making and the significant effect this can have on vaccine uptake [42,55], focusing only on country-level decisions. This national decision-making process is also unrealistic in assuming that every infection is equally costly (ignoring severe cases, deaths, hospital overload [56]), that discounting is constant over time (hyperbolic discounting is often considered the more realistic alternative [37,38]), and that the national agents are perfectly rational with full knowledge of the epidemiological and evolutionary processes at play. Prosociality is modeled in a highly simplified way, assuming that each country considers a fixed proportion of all outcomes from the other country in their utility function, combining policy mandates and psychology into one constant prosociality factor. And again, we assume the countries are identical in their degrees of discounting and prosociality, disregarding considerable variability in these factors between countries (especially between countries of different income levels [33]). The countries are different, however, in their ability to handle vaccine-distribution costs, with these costs being potentially burdensome to Country B but completely negligible to the higher-income Country A. Furthermore, it is possible that more efficient outcomes could be obtained by countries sharing commodities other than vaccines such as cash transfers in ways we have not considered in this paper.

On the other hand, an even simpler model without explicit consideration of epidemiological processes or continuous time may be able to reproduce some of the broader results that our modeling framework shows. However, a transmission model over continuous time is necessary for our novel method of calculating evolutionary potential over continuous time and to quantitatively gauge how epidemiological and evolutionary parameters affect the efficacy of vaccine sharing in a principled way.

## 4 Conclusion

Vaccine nationalism has featured prevalently in both major vaccine-preventable pandemics in recent history, namely the 2009 H1N1 and COVID-19 pandemics. However, we have previously lacked a framework to quantitatively assess the economic and behavioral drivers that underlie vaccine-hoarding tendencies [22], especially with consideration to viral evolution and the possibility of immune-escape variants. Using a novel framework combining epidemiological, evolutionary, and economic models, we show that the threat of future evolution and immune escape presents a problem of intertemporal choice for a vaccine-producing country, between hoarding vaccines to control their infections in the short term and sharing vaccines to control variant evolution and infections in the long term. As a result, a producing country’s discount rate prominently influences the amount of vaccines it will choose to donate to other countries in our modeling framework. We also explore how the optimal vaccine-sharing strategy is affected by prosociality towards other countries and development costs for vaccine-distribution infrastructure in receiving countries. Specifically, we conclude that vaccine hoarding is likely caused by a combination of high temporal discounting (either economically or psychologically induced), lack of prosociality, and high costs to receiving countries’ distribution infrastructure which are exacerbated by a lack of help from producing countries. Epidemiological and evolutionary parameters are also very important in determining a producing country’s optimal vaccine-sharing policy, as well as the means by which vaccine-sharing benefits the producing country: through an epidemiological benefit of controlling variant infections given variant emergence, or an evolutionary benefit of controlling the probability of variant emergence in the first place.

Research into the factors promoting vaccine nationalism is especially important in today’s age. The threat of HPAI spilling over from its current panzootic state in wild birds and poultry to become the next human pandemic looms large [13], as the effects of the severe vaccine nationalism fostered by the COVID-19 pandemic are still in recent memory. Thus, equitable vaccine distribution against HPAI may soon become an international priority, either for prophylaxis to prevent its spillover or for reducing infections and further evolution once spillover has occurred [57,58]. Furthermore, our work emphasizes the need for international organizations as central planners of global vaccine allocation in order to overcome the selfish priorities of vaccine-producing countries. However, at the time of this writing, the very humanitarian organizations that lead the global distribution of SARS-CoV-2 vaccines are under threat in the current political climate, following sweeping budget cuts to GAVI [14]. We hope this work can help advocate for the importance of such organizations and provide better knowledge of vaccine nationalism’s roots so we may prevent it from defining the next pandemic.

## 5 Methods

Our modeling framework consists of three submodels (Fig 1), each of which we describe briefly here and in more detail in S1 Appendix.

### 5.1 Epidemiological model

We consider two countries operating under SIR(S) dynamics, modifying the original SIRS model to have post-infection return to susceptibility marked by a transmissibility decrease for “secondary susceptibles” [49,59]. Specifically, we assume secondary susceptibles can be infected only by variant strains of the virus, not the wild-type, so that the wild-type strain effectively follows SIR dynamics with fully protective immunity, while variants follow SIRS dynamics with imperfect immunity (either due to strain replacement or reinfection by the same strain). In order to standardize the model’s behavior while varying the wild-type transmission rate $\beta_{w}$, the variant’s transmission rate against primary susceptibles $\beta_{v}$ is always increased (or decreased) 10% from $\beta_{w}$ and its transmission rate against secondary susceptibles is reduced by factor ϕ so that its reproduction number is scaled down to 1. This last assumption can be interpreted as individuals adopting interventions at just the right level to ensure the variants eventually die out [60]. The two countries’ dynamics are coupled by a small fraction η of interactions which happen between people of different countries and may lead to between-country transmissions, and by Country A sharing a fraction $f_{A}$ of its vaccine production rate ν with Country B.

### 5.2 Evolutionary model

We mechanistically measure the rate of variant emergence in each country based off the evolutionary potential formulae derived by [30]:


$$\begin{array}{cc} r_{A} & =\mu \beta_{w}S_{1,A}((1-\eta )I_{w,A}+\eta I_{w,B})\max\{1-\frac{\gamma }{\beta_{v}(S_{1,A}+\phi S_{2,A})},0\} \end{array}$$ (1)

$$\begin{array}{cc} r_{B} & =\mu \beta_{w}S_{1,B}((1-\eta )I_{w,B}+\eta I_{w,A})\max\{1-\frac{\gamma }{\beta_{v}(S_{1,B}+\phi S_{2,B})},0\}, \end{array}$$ (2)

with *S*_1,*X*_, *S*_2,*X*_, and $I_{w}$ representing primary susceptibles, secondary susceptibles, and wild-type infections, respectively, in Country X (A or B); μ is the per-transmission mutation rate from wild-type to variant; and γ is the infection recovery rate. From these, the probability $p^{w}$ that the variant has not yet emerged follows $\frac{dp^{w}}{dt}=-(r_{A}+r_{B})p^{w}$, and the instantaneous probability that the variant emerges in Country X (A or B) at time *t* is $r_{X}(t)p^{w}(t)dt$.

### 5.3 Economic model

We calculate the instantaneous infection costs for Country X (A or B) at time *t*, $J_{X}(t)$, as the weighted average of the wild-type/variant incidence in Country X at time *t* in all possible scenarios of variant emergence: no variant has emerged (weighted wi*t*h probability $p^{w}(t)$), the variant emerged in Country A at time τ (probability $r_{A}(\tau )p^{w}(\tau )dt$), or the variant emerged in Country B at time τ (probability $r_{B}(\tau )p^{w}(\tau )dt$). The total infection cost for Country A from present time *t* = 0 until some maximum time *t* = *T*, with temporal discounting at rate δ and prosociality factor ρ, is then


$$\begin{array}{c} C_{A}=\int_{0}^{T}e^{-\delta t}[(1-\rho )J_{A}(t)+\rho J_{B}(t)]dt, \end{array}$$ (3)

and a similar equation with *A* and *B* switched gives the total infection cost for Country B. Country A is trying to minimize its infection costs $C_{A}$ by sharing the optimal fraction $f_{A}^{*}$ of its vaccines. In later sections, we allow Country B to simultaneously decide the maximum fraction $f_{B}$ of vaccines it is able to accept based on an added cost $c(f_{B})$ to developing a sufficient vaccine-distribution infrastructure. In this case, Country B’s vaccination rate becomes $\min\{f_{A},f_{B}\}\nu$, while Country A’s vaccination rate remains $(1-f_{A})\nu$.

## Supporting information

S1 Appendix Detailed model description, mathematical proofs, additional analyses, and supplement figures. (PDF)
