# Supplementary material for: Economic factors promoting vaccine nationalism in the face of viral evolution
Source: PLoS Comput Biol. 2026 Aug 31;22(8):e1014466. doi: 10.1371/journal.pcbi.1014466 (PMC13541263; doi:10.1371/journal.pcbi.1014466)
Supplement: Supplementary file 1 [file pcbi.1014466.s001.pdf]

Supporting Information for:
Economic factors promoting vaccine nationalism in the face of viral evolution

Ari S. Freedman[1,2,3*], Bjarke Frost Nielsen[4,5,6], Chadi M. Saad-Roy[7,8,9,10,11], Bryan T. Grenfell[1], C. Jessica E. Metcalf[1], Simon A. Levin[1]

[1]Department of Ecology and Evolutionary Biology, Princeton University, Princeton, New Jersey, United States of America

[2]Department of Plant Biology, University of Vermont, Burlington, Vermont, United States of America

[3]Vermont Complex Systems Institute, University of Vermont, Burlington, Vermont, United States of America

[4]High Meadows Environmental Institute, Princeton University, Princeton, New Jersey, United States of America

[5]Niels Bohr Institute, University of Copenhagen, Copenhagen, Denmark

[6]PandemiX Center, Roskilde University, Roskilde, Denmark

[7]Miller Institute for Basic Research in Science, University of California, Berkeley, California, United States of America

[8]Department of Integrative Biology, University of California, Berkeley, California, United States of America

[9]Department of Mathematics, The University of British Columbia, Vancouver, Canada

[10]Department of Department of Microbiology and Immunology, The University of British Columbia, Vancouver, Canada

[11]Biodiversity Research Centre, University of British Columbia, Vancouver, Canada

*arisf@stanford.edu

Contents

1 Modeling framework **3**

1.1 Epidemiological Model . . . 3

1.2 Evolutionary Model . . . 4

1.3 Economic model . . . 5

1.4 Discretizing variant emergence times . . . 6

1.5 Variable/parameter symbols and default values . . . 8

1.6 Epidemiological model equations . . . 9

1.6.1 ODEs with no variant . . . 9

1.6.2 ODEs with variant . . . 9

2 Analytic results about the effect of distribution costs on vaccine sharing Nash equilibria **11**

3 Supplementary figures and results **18**

3.1 Example with both country's dynamics and susceptibilities . . . 18

3.2 Example with low wild-type $R_0 = 1.5$ (vaccine sharing has evolutionary benefit instead of epidemiological benefit) . . . 19

3.3 Variant emergence probabilities by country and mutation rate . . . 21

3.4 Variant has 10% transmission decrease (instead of 10% increase) . . . 23

3.5 More vaccine distribution cost functions $c(f_B)$ . . . 24

3.6 Country A helps with Country B's infrastructure costs . . . 25

1 Modeling framework

1.1 Epidemiological Model

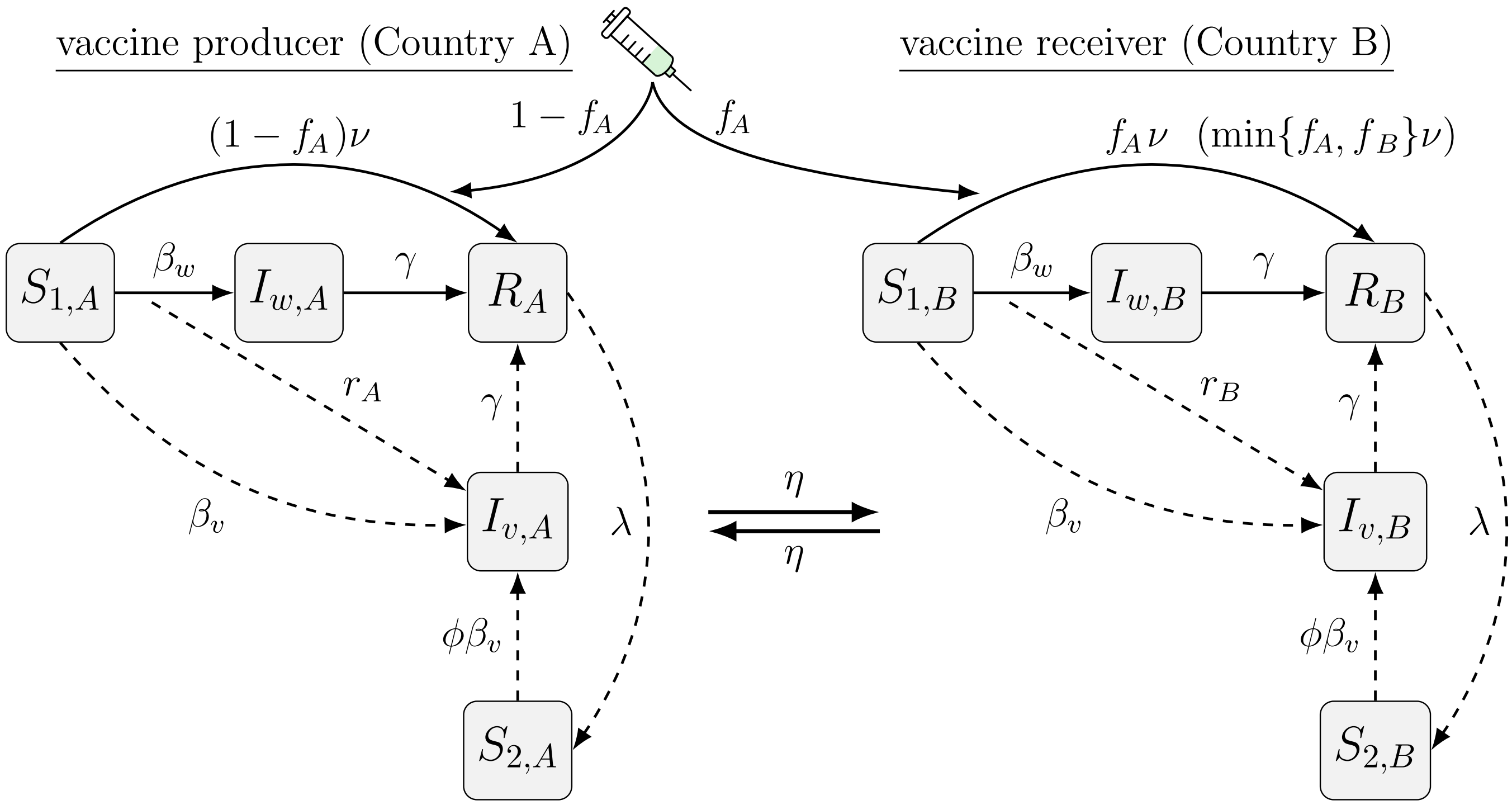

Figure A: Compartmental diagram representing the ordinary differential equations that underlie the epidemiological model. Both Country A (subscripts A) and Country B (subscripts B) have SIR(S) infection dynamics. The two countries are coupled through a fraction η of interactions which occur between individuals of different countries, and through Country A sharing a fraction f_A of its vaccine production rate ν so that Country A's vaccination rate is $(1 - f_A)\nu$ and Country B's is $f_A\nu$ (or $\min\{f_A, f_B\}\nu$ if Country B sets a cap f_B on the share of vaccines it can accept, as we add later).

In order to explore how these various factors shape a vaccine producing country's vaccine allocation strategy, we employ an SIR(S) epidemiological model, which modifies the original SIRS model to have post-infection return to susceptibility marked by a decrease in transmissibility for "secondary susceptibles" [1, 2]. Specifically, we assume secondary susceptibles can be infected only by variant strains of the virus, not the wild-type, so that the wild-type strain effectively follows SIR dynamics with fully protective immunity. Fig. A depicts the structure of the ordinary differential equations that underlie our epidemiological model: primary susceptible individuals (S_1) can get infected either by wild-type infecteds (I_w) at rate β_w or by variant infecteds (I_v) at rate β_v, after which infectiousness wanes at rate γ to take them to the recovered class (R) and immunity wanes

at rate λ to take them to the secondary susceptible class (S_2), where they are susceptible only to variant infections at reduced rate $\phi\beta_v$ (with ϕ scaling the variant's reproduction number against secondary susceptibles to 1 to standardize the model's long-term behavior). Primary susceptible individuals may also be vaccinated, taking them straight to the recovered class.

The two countries are coupled through a fraction η of interactions which occur between individuals of different countries, and through Country A sharing a fraction f_A of its vaccine production rate ν so that Country A's vaccination rate is $(1 - f_A)\nu$ and Country B's is $f_A\nu$ (or $\min\{f_A, f_B\}\nu$ if Country B sets a cap f_B on the share of vaccines it can accept, as we add later).

Full model equations are given in Section 1.6 and parameter values in Section 1.5.

1.2 Evolutionary Model

In order to capture how variant emergence depends on population-level prevalence and landscape of immunity, we mechanistically derive a time-varying net viral adaptation rate for each country, following [3]:

$$r_A = \mu\beta_w S_{1,A}\big((1-\eta)I_{w,A} + \eta I_{w,B}\big) \max\left\{1 - \frac{\gamma}{\beta_v(S_{1,A} + \phi S_{2,A})}, 0\right\} \quad (1)$$

$$r_B = \mu\beta_w S_{1,B}\big((1-\eta)I_{w,B} + \eta I_{w,A}\big) \max\left\{1 - \frac{\gamma}{\beta_v(S_{1,B} + \phi S_{2,B})}, 0\right\}, \quad (2)$$

where μ is the per-transmission mutation rate from wild-type to immune-escape variant scaled by the population size, and subscripts of A or B specifies country. The first parts of these expressions, $\mu\beta_w S_{1,A}\big((1-\eta)I_{w,A} + \eta I_{w,B}\big)$ and $\mu\beta_w S_{1,B}\big((1-\eta)I_{w,B} + \eta I_{w,A}\big)$, represent the rates at which an escape variant can currently evolve in each country based on the number of transmissions currently occurring in each country. These are simply μ times the instantaneous incidence of new wild-type infections in each country. The second parts, $\max\left\{1 - \frac{\gamma}{\beta_v(S_{1,A}+\phi S_{2,A})}, 0\right\}$ and $\max\left\{1 - \frac{\gamma}{\beta_v(S_{1,B}+\phi S_{2,B})}, 0\right\}$, represent the probabilities that a newly evolved escape variant in each country will be able to establish itself without dying from stochastic extinction, based on the susceptibility profiles of each country and the assumption that the initial growth of a new variant can be modeled by a simple branching process. See [3] for a full derivation.

Given these time-varying viral adaptation rates r_A and r_B, the probabilities that by time t a

variant has emerged in Country A ($p^A(t)$), has emerged in Country B ($p^B(t)$) or has not emerged ($p^w(t)$) can then be traacked by

$$\frac{dp^A}{dt} = r_A p^w \tag{3}$$
$$\frac{dp^B}{dt} = r_B p^w \tag{4}$$
$$\frac{dp^w}{dt} = -(r_A + r_B)p^w \tag{5}$$

with $p_A(0) = p_B(0) = 0$ and $p_w(0) = 1$; and the derivatives $\frac{dp^A}{dt}$ and $\frac{dp^B}{dt}$ can be thought of as the instantaneous probabilities of variant emergence in each country.

1.3 Economic model

We now describe each country's infection costs, from which they base their vaccine-related policy decisions. We assume only one variant strain may emerge, but explore parallel scenarios in which the variant emerges at different points in time τ. The cost of infection at a specific time t for each country is then a weighted average of the total incidence at time t in all possible scenarios: no variant has emerged (probability $p^w(t)$), the variant emerged in Country A at time τ (probability $\frac{dp^A}{dt}(\tau)$) for all possible $\tau < t$, or the variant emerged in Country B at time τ (probability $\frac{dp^B}{dt}(\tau)$) for all $\tau < t$. The instantaneous infection costs for Country A and Country B are then

$$J_A(t) = p^w(t) i^w_{w,A}(t) + \int_0^t \left[\tfrac{dp^A}{d\tau}(\tau)\big(i^{A,\tau}_{w,A}(t) + i^{A,\tau}_{v,A}(t)\big) + \tfrac{dp^B}{d\tau}(\tau)\big(i^{B,\tau}_{w,A}(t) + i^{B,\tau}_{v,A}(t)\big)\right] d\tau \tag{6}$$
$$J_B(t) = p^w(t) i^w_{w,B}(t) + \int_0^t \left[\tfrac{dp^A}{d\tau}(\tau)\big(i^{A,\tau}_{w,B}(t) + i^{A,\tau}_{v,B}(t)\big) + \tfrac{dp^B}{d\tau}(\tau)\big(i^{B,\tau}_{w,B}(t) + i^{B,\tau}_{v,B}(t)\big)\right] d\tau \tag{7}$$

where superscripts denote the scenario from which the infections were simulated (w for no variant emergence, A or B for the country of emergence, and τ for time of emergence), and lowercase i represents instantaneous incidence (as opposed to capital I which represents prevalence). For example, $i^{A,\tau}_{w,B}(t)$ refers to the instantaneous incidence of wild type infections in Country B at time t given that a variant emerged in Country A at time τ, and $i^{A,\tau}_{v,B}(t)$ is the instantaneous incidence of variant infections in Country B at time t given that a variant emerged in Country A at time τ.

Finally, the total infection cost for Country A, with temporal discounting at rate δ, prosociality

factor ρ, and maximum time of consideration T, is then

$$C_A = \int_0^T e^{-\delta t} \Big[(1-\rho) J_A(t) + \rho J_B(t) \Big] dt. \tag{8}$$

In our initial analyses in the main text, only Country A's infection cost is relevant, as they are the ones who decide the vaccine sharing policy governed by f_A, the fraction of vaccines they we will share with Country B.

We later consider decision agency in Country B as well, who chooses a cap f_B to the fraction of vaccines they are willing to accept based on high costs $c(f_B)$ to developing a suitably sized vaccine distribution infrastructure. The choice of f_B then affects the other models by changing Country B's vaccination rate from $f_A \nu$ to $\min\{f_A, f_B\}\nu$. The distribution cost $c(f_B)$ is then added to their infection cost to give a total cost for Country B

$$C_B = \int_0^T e^{-\delta t} \Big[(1-\rho) J_B(t) + \rho J_A(t) \Big] dt + c(f_B). \tag{9}$$

Country B is trying to minimize their total cost C_B based on their choice of vaccine distribution cap f_B, while Country A is trying to minimize their total cost C_A based on their choice of vaccine-sharing fraction f_A.

The distribution cost function can be anything with $c(0) = 0$ and $c'(f_B) > 0$ for all $0 < f_B < 1$. In our simulations we only consider linear $c(f_B) = \alpha f_B$ for some $\alpha \geq 0$; but in our analytic results in Section 2, we consider more general possibilities for $c(f_B)$.

1.4 Discretizing variant emergence times

To make simulating this modeling framework computationally feasible, we necessary to discretize the possible times at which variants may emerge, assuming variants may only emerge at discrete timesteps τ_k (we choose timesteps of length $\tau_k - \tau_{k-1} = 1$ week) and replacing some of the integrals over time in Eqs. (6)–(9) with sums over timesteps.

In this approximation, the cost of infection during the k-th timestep ($\tau_{k-1} < t < \tau_k$) is now a weighted average of the total incidence at time t in all possible scenarios: no variant has yet emerged by the beginning of the timestep (probability $p^w(\tau_{k-1})$), the variant emerged in Country A in a previous j-th timestep (probability $p^A(\tau_j) - p^A(\tau_{j-1})$) for all $j < k$, or the variant emerged in

Country B in a previous j-th timestep (probability $p^B(\tau_j) - p^B(\tau_{j-1})$) for all $j < k$. The infection costs during timestep k for Country A and Country B are now

$$
\begin{aligned}
J_A(\tau_k) = \int_{\tau_{k-1}}^{\tau_k} \Bigg[p^w(\tau_{k-1}) i^w_{w,A}(t) + & \sum_{\substack{0<\tau_j<\tau_k \\ p^A(\tau_j)-p^A(\tau_{j-1})>\varepsilon}} \left(p^A(\tau_j) - p^A(\tau_{j-1})\right)\left(i^{A,\tau_j}_{w,A}(t) + i^{A,\tau_j}_{v,A}(t)\right) + \\
& \sum_{\substack{0<\tau_j<\tau_k \\ p^B(\tau_j)-p^B(\tau_{j-1})>\varepsilon}} \left(p^B(\tau_j) - p^B(\tau_{j-1})\right)\left(i^{B,\tau_j}_{w,A}(t) + i^{B,\tau_j}_{v,A}(t)\right)\Bigg] dt
\end{aligned} \tag{10}
$$

$$
\begin{aligned}
J_B(\tau_k) = \int_{\tau_{k-1}}^{\tau_k} \Bigg[p^w(\tau_{k-1}) i^w_{w,B}(t) + & \sum_{\substack{0<\tau_j<\tau_k \\ p^A(\tau_j)-p^A(\tau_{j-1})>\varepsilon}} \left(p^A(\tau_j) - p^A(\tau_{j-1})\right)\left(i^{A,\tau_j}_{w,B}(t) + i^{A,\tau_j}_{v,B}(t)\right) + \\
& \sum_{\substack{0<\tau_j<\tau_k \\ p^B(\tau_j)-p^B(\tau_{j-1})>\varepsilon}} \left(p^B(\tau_j) - p^B(\tau_{j-1})\right)\left(i^{B,\tau_j}_{w,B}(t) + i^{B,\tau_j}_{v,B}(t)\right)\Bigg] dt,
\end{aligned} \tag{11}
$$

replacing Eqs. (6)–(7), where again superscripts denote the scenario from which the infections were simulated (w for no variant emergence, A or B for the country of emergence, and τ_j for timestep of emergence), and lowercase i represents instantaneous incidence (as opposed to capital I which represents prevalence). Note that we set a lower limit ε to the viral adaptation rate in a timestep that will actually lead to an emergence scenario in the next timestep; however, by making ε sufficiently small, this will not change the results noticeably, as low-probability emergence scenarios contribute little to the total weighted incidence (we set $\varepsilon = 10^-6$, a very low probability for an event to happen in a week).

The total infection costs for Country A and Country B, with discounting and prosociality taken into account, are now

$$C_A = \sum_{k=1}^{K} e^{-\delta\tau_k} \left((1-\rho)J_A(\tau_k) + \rho J_B(\tau_k)\right) \tag{12}$$

$$C_B = \sum_{k=1}^{K} e^{-\delta\tau_k} \left((1-\rho)J_B(\tau_k) + \rho J_A(\tau_k)\right) + c(f_B), \tag{13}$$

replacing Eqs. (8)–(9), and with $\tau_K = T$ being the maximum time of consideration.

1.5 Variable/parameter symbols and default values

Symbol	Meaning		
$S_{1,X}$	Primary susceptible individuals in Country X		
$S_{2,X}$	Secondary susceptible individuals in Country X		
$I_{w,X}$	People infected with wild-type virus in Country X		
$I_{v,X}$	People infected with variant virus in Country X		
R_X	Recovered, temporarily immune individuals in Country X		
^{w}	Superscript denoting scenario in which variant has not yet emerged		
$^{X,\tau}$	Superscript denoting scenario in which variant emerged in Country X in time(step) τ		
**Symbol**	**Meaning**	**Value**	**Range**
N	Population size of each country	10^6	
R_0	Wild-type basic reproduction number	2	1–3
β_w	Transmission rate for wild-type virus	$R_0\gamma$	
β_v	Transmission rate for variant virus	$1.1\beta_w$	$.9\beta_w$, $1.1\beta_w$
γ	Daily disease recovery rate	1/10	
λ	Annual rate of loss of immunity to variants	2	
ϕ	Reduction in susceptibility for secondary infections	γ/β_v	
η	Fraction of interactions occurring between people of different countries	.05	0–.2
ν	Annual per-person rate at which Country A produces vaccines	1	.5–2.5
f_A	Fraction of vaccine production Country A shares with Country B		0–1
f_B	Maximum fraction of vaccine production Country B is able to distribute	1	0–1

Table A: Variables and parameters from the epidemiological submodel, along with default values that each parameter takes unless stated otherwise.

Symbol	Meaning		
r_X	Viral adaptation rate of immune-escape variant emergence in Country X		
$i_{w,X}$	Instantaneous incidence of wild-type infections virus in Country X		
$i_{v,X}$	Instantaneous incidence of variant infections virus in Country X		
J_X	New infection costs at specific time or during specific timestep in Country X		
C_X	Total costs for Country X		
f_A^*	Optimal fraction f_A of vaccine production for Country A to share to minimize C_A		
**Symbol**	**Meaning**	**Default**	**Range**
μ	Population-scaled daily mutation rate	2	10^{-1}–10^3
δ	Annual temporal discount rate	.05	.001–.2
ρ	Prosociality factor (fraction of other country's costs that a country considers)	.05	0–.5
$c(f_B)$	Cost to Country B to developing vaccine distribution infrastructure	αf_B	
α	Cost of distributing one vaccine as compared to one infection	0	0, 1, 2, 4
T	Population size of each country	10^6	
ε	Minimum total viral adaptation rate over timestep required for variant emergence	10^{-6}	
$\tau_k - \tau_{k-1}$	Timestep length	1 week	
ζ	Fraction of Country B's infrastructure costs that Country A pays for	0	0–1
x	Reduction in costs for Country A to pay for infrastructure compared to Country B	1	.25, .5, 1

Table B: Other variables and parameters, along with default values that each parameter takes unless stated otherwise.

1.6 Epidemiological model equations

1.6.1 ODEs with no variant

$$\frac{dS^w_{1,A}}{dt} = -\beta_w((1-\eta)I^w_{w,A} + \eta I^w_{w,B})S^w_{1,A} - (1-f_A)\nu S^w_{1,A} \tag{14}$$
$$\frac{dS^w_{2,A}}{dt} = \lambda R^w_A \tag{15}$$
$$\frac{dI^w_{w,A}}{dt} = \beta_w((1-\eta)I^w_{w,A} + \eta I^w_{w,B})S^w_{1,A} - \gamma I^w_{w,A} - \mu I^w_{w,A} \tag{16}$$
$$\frac{dR^w_A}{dt} = \gamma I^w_{w,A} - \lambda R^w_A + (1-f_A)\nu S^w_{1,A} \tag{17}$$

$$\frac{dS^w_{1,B}}{dt} = -\beta_w((1-\eta)I^w_{w,B} + \eta I^w_{w,A})S^w_{1,B} - \min\{f_A, f_B\}\nu S^w_{1,B} \tag{18}$$
$$\frac{dS^w_{2,B}}{dt} = \lambda R^w_B \tag{19}$$
$$\frac{dI^w_{w,B}}{dt} = \beta_w((1-\eta)I^w_{w,B} + \eta I^w_{w,A})S^w_{1,B} - \gamma I^w_{w,B} - \mu I^w_{w,B} \tag{20}$$
$$\frac{dR^w_B}{dt} = \gamma I^w_{w,B} - \lambda R^w_B + \min\{f_A, f_B\}\nu S^w_{1,B} \tag{21}$$

with initial conditions $S^w_{1,X}(0) = 1 - \frac{1}{N}$, $I^w_{w,X}(0) = \frac{1}{N}$, $p^w(0) = 1$, and $S^w_{2,X}(0) = R^w_X(0) = p^X(0) = 0$, where X is either A or B. N is population size, which we set to be the same for both countries.

1.6.2 ODEs with variant

ODEs with variant arising in country Y (either A or B) at the i-th timestep $\tau_i > 0$:

$$\begin{aligned}\frac{dS^{Y,\tau_i}_{1,A}}{dt} = &-\beta_w((1-\eta)I^{Y,\tau_i}_{w,A} + \eta I^{Y,\tau_i}_{w,B})S^{Y,\tau_i}_{1,A} \\ &-\beta_v((1-\eta)I^{Y,\tau_i}_{v,A} + \eta I^{Y,\tau_i}_{v,B})S^{Y,\tau_i}_{1,A} - (1-f_A)\nu S^{Y,\tau_i}_{1,A}\end{aligned} \tag{22}$$
$$\frac{dS^{Y,\tau_i}_{2,A}}{dt} = \lambda R^{Y,\tau_i}_A - \phi\beta_v((1-\eta)I^{Y,\tau_i}_{v,A} + \eta I^{Y,\tau_i}_{v,B})S^{Y,\tau_i}_{2,A} \tag{23}$$
$$\frac{dI^{Y,\tau_i}_{w,A}}{dt} = \beta_w((1-\eta)I^{Y,\tau_i}_{w,A} + \eta I^{Y,\tau_i}_{w,B})S^{Y,\tau_i}_{1,A} - \gamma I^{Y,\tau_i}_{w,A} - \mu I^{Y,\tau_i}_{w,A} \tag{24}$$
$$\frac{dI^{Y,\tau_i}_{v,A}}{dt} = \beta_v((1-\eta)I^{Y,\tau_i}_{v,A} + \eta I^{Y,\tau_i}_{v,B})(S^{Y,\tau_i}_{1,A} + \phi S^{Y,\tau_i}_{2,A}) - \gamma I^{Y,\tau_i}_{v,A} + \mu I^{Y,\tau_i}_{w,A} \tag{25}$$
$$\frac{dR^{Y,\tau_i}_A}{dt} = \gamma(I^{Y,\tau_i}_{w,A} + I^{Y,\tau_i}_{v,A}) - \lambda R^{Y,\tau_i}_A + (1-f_A)\nu S^{Y,\tau_i}_{1,A} \tag{26}$$

$$\frac{dS_{1,B}^{Y,\tau_i}}{dt} = -\beta_w((1-\eta)I_{w,B}^{Y,\tau_i} + \eta I_{w,A}^{Y,\tau_i})S_{1,B}^{Y,\tau_i} \\ -\beta_v((1-\eta)I_{v,B}^{Y,\tau_i} + \eta I_{v,A}^{Y,\tau_i})S_{1,B}^{Y,\tau_i} - \min\{f_A, f_B\}\nu S_{1,B}^{Y,\tau_i} \tag{27}$$

$$\frac{dS_{2,B}^{Y,\tau_i}}{dt} = \lambda R_B^{Y,\tau_i} - \phi\beta_v((1-\eta)I_{v,B}^{Y,\tau_i} + \eta I_{v,A}^{Y,\tau_i})S_{2,B}^{Y,\tau_i} \tag{28}$$

$$\frac{dI_{w,B}^{Y,\tau_i}}{dt} = \beta_w((1-\eta)I_{w,B}^{Y,\tau_i} + \eta I_{w,A}^{Y,\tau_i})S_{1,B}^{Y,\tau_i} - \gamma I_{w,B}^{Y,\tau_i} - \mu I_{w,B}^{Y,\tau_i} \tag{29}$$

$$\frac{dI_{v,B}^{Y,\tau_i}}{dt} = \beta_v((1-\eta)I_{v,B}^{Y,\tau_i} + \eta I_{v,A}^{Y,\tau_i})(S_{1,B}^{Y,\tau_i} + \phi S_{2,B}^{Y,\tau_i}) - \gamma I_{v,B}^{Y,\tau_i} + \mu I_{w,B}^{Y,\tau_i} \tag{30}$$

$$\frac{dR_B^{Y,\tau_i}}{dt} = \gamma(I_{w,B}^{Y,\tau_i} + I_{v,B}^{Y,\tau_i}) - \lambda R_B^{Y,\tau_i} + \min\{f_A, f_B\}\nu S_{1,B}^{Y,\tau_i} \tag{31}$$

with initial conditions at $t = \tau_i$ ($t < \tau_i$ is irrelevant as the no-variant ODEs describe what happens pre-variant emergence) of

$$S_{1,X}^{Y,\tau_i}(\tau_i) = \begin{cases} S_{1,X}^w(\tau_i) - \frac{1}{N} & \text{if } X = Y \\ S_{1,X}^w(\tau_i) & \text{if } X \neq Y \end{cases} \tag{32}$$

$$I_{v,X}^{Y,\tau_i}(\tau_i) = \begin{cases} \frac{1}{N} & \text{if } X = Y \\ 0 & \text{if } X \neq Y \end{cases} \tag{33}$$

$$S_{2,X}^{Y,\tau_i}(\tau_i) = S_{2,X}^w(\tau_i) \tag{34}$$

$$I_{w,X}^{Y,\tau_i}(\tau_i) = I_{w,X}^w(\tau_i) \tag{35}$$

$$R_X^{Y,\tau_i}(\tau_i) = R_X^w(\tau_i) \tag{36}$$

where X and Y are both either A or B. In other words, emergence is assumed to happen during transmission (as also reflected by the viral adaptation rate equations (1)–(2)), after which one person is suddenly infected with the variant and one less person is susceptible (namely the person who got infected with the first variant infection).

2 Analytic results about the effect of distribution costs on vaccine sharing Nash equilibria

In this section, we give a criterion for when the game between Country A and Country B who choose strategies in the form of f_A and f_B, respectively, will result in Nash equilibria that are all suboptimal for Country A. We thus seek to better explain our game-theoretic results presented in main text Fig. 6 and Fig. J.

We generalize here away from vaccine sharing to Country A sharing some resource with Country B at level f_A (ranging from 0 to 1), while Country B chooses a maximum level f_B (also ranging from 0 to 1) of the resource it can accept given that it has additional costs $c(f_B)$ to develop infrastructure to properly use the resource at that level. Country A and Country B both have costs that they are trying to minimize: without infrastructure costs, these costs are $C_A^I(f_A, f_B)$ and $C_B^I(f_A, f_B)$, respectively, which are both functions of f_A and f_B (superscript I stands for infection costs, though in this more generalized scenario the costs need not be due to infection). We assume that Country A sharing the resource minimizes its cost at some non-zero level f_A^*, assuming Country B is able to use all of that resource ($f_B = 1$). We codify these and a few other assumptions about these cost functions here:

$$C_A^I(f_A, f_B) \text{ and } C_B^I(f_A, f_B) \text{ are continuously differentiable and positive} \tag{37}$$

$$C_A^I(f_A, f_B) = C_A^I(f_A, f_A) \text{ and } C_B^I(f_A, f_B) = C_B^I(f_A, f_A)\ \forall f_A \leq f_B \tag{38}$$

$$\text{The function } \psi(f_A) := C_I^A(f_A, 1) \text{ has exactly one local minimum } 0 < f_A^* < 1 \tag{39}$$

$$\frac{\partial C_A^I(f_A, f_B)}{\partial f_A} > 0\ \forall f_A > f_B \text{ (bad for Country A to share resource that goes unused)} \tag{40}$$

$$\frac{\partial C_B^I(f_A, f_B)}{\partial f_B} < 0\ \forall f_B < f_A \text{ (bad for Country B to use less resource than shared)} \tag{41}$$

$$\frac{\partial^2 C_B^I(f_A, f_B)}{\partial f_B^2} > 0\ \forall f_B < f_A \text{ (worse for Country B to waste resource when it has less)} \tag{42}$$

$$g(f_A) := -\frac{\partial^-}{\partial f_B} C_B^I(f_A, f_B)\bigg|_{f_B = f_A} \text{ satisfies } g(f_A) > 0 \text{ and } g'(f_A) < 0\ \forall f_A \tag{43}$$

(less costly for Country B to reject resource when given more resource).

We can then apply our vaccine sharing model defined in (6)–(9) to this generalized resource sharing framework by plugging in C_A and C_B from (8)–(9) into $C_A^I(f_A, f_B)$ and $C_B^I(f_A, f_B)$, respectively. These C_A and C_B satisfy all of the assumptions (37)–(43), as we show for assumptions (42)–(43) in Figs. B–C.

Their total costs with infrastructure development accounted for are then

$$C_A(f_A, f_B) = C_A^I(f_A, f_B) \tag{44}$$

$$C_B(f_A, f_B) = C_B^I(f_A, f_B) + c(f_B), \tag{45}$$

where Country A has no additional costs but Country B has an additional infrastructure cost $c(f_B)$ that is continuously differentiable and satisfies $c(0) = 0$, $c'(f_B) \geq 0$, and $c''(f_B) \geq 0$ for all f_B. These total cost functions $C_A(f_A, f_B)$ and $C_B(f_A, f_B)$ are what Country A and Country B, respectively, are trying to minimize through each country simultaneously and independently choosing a value of f_A or f_B, respectively. By definition, a strategy profile of $(\hat{f}_A, \hat{f}_B)$ will be a Nash equilibrium if $C_A(\hat{f}_A, \hat{f}_B) \leq C_A(f_A, \hat{f}_B)\ \forall f_A$ and $C_B(\hat{f}_A, \hat{f}_B) \leq C_A(\hat{f}_A, f_B)\ \forall f_B$, or equivalently, if $\hat{f}_A \in \underset{0 \leq f_A \leq 1}{\arg\min}\, C_A(f_A, \hat{f}_B)$ and $\hat{f}_B \in \underset{0 \leq f_B \leq 1}{\arg\min}\, C_A(\hat{f}_A, f_B)$ (where arg min is a set-valued function returning all argument that produce equal minima) [4].

We now work up to the main result, which shows that there will exist a Nash equilibrium $(\hat{f}_A, \hat{f}_B)$ where $\hat{f}_A = f_A^*$ — meaning Country A gets to share the level of resource that is optimal for it — if and only if $g(f_A^*) \geq c'(f_A^*)$, with $g(f_A)$ defined by (43); and if $g(f_A^*) < c'(f_A^*)$, then $\hat{f}_A < f_A^*$ for all Nash equilibria $(\hat{f}_A, \hat{f}_B)$ — meaning Country A will end up sharing less of the resource than is optimal for it due to the high costs of properly using the resource in Country B. And in the case where the cost function $c(f_B) = \alpha f_B$ is linear (as in our simulations), this statement can be slightly simplified by substituting with $c'(f_A) = \alpha$ so that the condition of interest is whether $g(f_A^*) \geq \alpha$ or $g(f_A^*) < \alpha$.

We first establish the forms of the cost-minimizing conditional strategies for each Country (the black and gray curves in main text Fig. 6 and Fig. J).

Lemma 1.

$$\underset{0 \leq f_A \leq 1}{\arg\min}\, C_A(f_A, f_B) = \begin{cases} \{f_B\}, & f_B < f_A^* \\ \{f_A^*\}, & f_B \geq f_A^* \end{cases} \tag{46}$$

$$\underset{0 \leq f_B \leq 1}{\arg\min}\, C_B(f_A, f_B) = \begin{cases} \{f_A\}, & c(f_A) > 0 \text{ and } g(f_A) \geq c'(f_A) \\ \{< f^A\}, & c(f_A) > 0 \text{ and } g(f_A) < c'(f_A) \\ \{f_B \mid f_B \geq f_A \text{ and } c(f_B) = 0\} & c(f_A) = 0, \end{cases} \tag{47}$$

where the second case of (47) says that there is one value of f_B for which $C_B(f_A, f_B)$ achieves a minimum (with f_A fixed) and that value of f_B is less than f_A.

Proof. Note that (39) implies $\psi(f_A)$ is decreasing for $f_A < f_A^*$ and increasing for $f_A > f_A^*$, and (44) and (39) imply

$$C_A(f_A, f_B) = \psi(f_A)\ \forall f_A \leq f_B. \tag{48}$$

Thus, if $f_B < f_A^*$: then $C_A(f_B, f_B) = \psi(f_B) < \psi(f_A) = C_A(f_A, f_B)$ for any $f_A < f_B$; and $C_A(f_B, f_B) < C_A(f_A, f_B)$ for any $f_A > f_B$ by (40). This proves the first case of (46).

Similarly, if $f_B \geq f_A^*$: then $C_A(f_A^*, f_B) = \psi(f_A^*) < \psi(f_B) = C_A(f_A, f_B)$ for any $f_A \leq f_B$ with $f_A \neq f_A^*$; and for $f_A > f_B$, (40) implies $C_A(f_A^*, f_B) < C_A(f_B, f_B) < C_A(f_A, f_B)$. This proves the second case of (46).

We now prove the third case of (47). Fix a value of f_A with $c(f_A) = 0$, and let $X = \{f_B \mid f_B \geq f_A \text{ and } c(f_B) = 0\}$, which is non-empty since it includes f_A. Since $c(f_B)$ is continuous and non-decreasing, X will be a closed interval $\{f_B \mid f_A \geq f_B \geq m\}$ for some $m \geq f_A$ that satisfies $c(f_B) = 0\ \forall f_B \leq m$. Now (38) implies that $C_B(f_A, f_B) = C_B^I(f_A, f_B)$ is constant over all $f_B \in X$, and that $C_B(f_A, f_A) = C_B^I(f_A, f_B) < C_B(f_A, f_B)$ for all $f_B > m$ (since $c(f_B) > 0$ for $f_B > m$). Furthermore, (41) implies that $C_B(f_A, f_A) < C_B(f_A, f_B)$ for all $f_B < f_A$. Combining these facts gives us that the set on which $C_B(f_A, f_B)$ achieves a minimum is X.

With the third case of (47) done and keeping f_A fixed, we can now assume $c(f_A) > 0$, and thus $c(f_B) > 0$ and $c'(f_B) > 0$ for all $f_B \geq f_A$ (since $c(0) = 0$ and $c''(f_B) \geq 0$ for all f_B). We then have that $C_B(f_A, f_B) = C_B^I(f_A, f_A)$ is strictly increasing in f_B for $f_B \geq f_A$ by (38), and so any f_B on which $C_B(f_A, f_B)$ achieves a minimum must satisfy $f_B \leq f_A$. Furthermore, $C_B(f_A, f_B)$ must

achieve a minimum at exactly one value of $f_B \leq f_A$, since

$$\frac{\partial^2 C_B(f_A, f_B)}{\partial f_B^2} = \frac{\partial^2 C_B^I(f_A, f_B)}{\partial f_B^2} + c''(f_B) > 0 \ \forall f_B < f_A \tag{49}$$

by (42). If $g(f_A) \geq c'(f_A)$, then $\frac{\partial C_B(f_A,f_B)}{\partial f_B}\Big|_{f_B=f_A} = \frac{\partial C_B^I(f_A,f_B)}{\partial f_B}\Big|_{f_B=f_A} + c'(f_A) \leq 0$, which combined with (49) tells us that $C_B(f_A, f_B)$ will achieve this minimum at $f_B = f_A$. And if $g(f_A) < c'(f_A)$, then $\frac{\partial C_B(f_A,f_B)}{\partial f_B}\Big|_{f_B=f_A} > 0$, which tells us that $C_B(f_A, f_B)$ will achieve this minimum at a value of f_B that is strictly less than f_A.

$\square$

We now prove the main theorem characterizing the Nash equilibria set and a corollary showing how this predicts when Country A is forced to share less resource than is optimal for it.

Theorem 1. *Exactly one of these three cases will be true:*

1. *If* $g(0) \leq c'(0)$*, then the only Nash equilibrium is* $(0, 0)$*.*
2. *If* $g(f_A^*) \geq c'(f_A^*)$*, then the set of Nash equilibria is* $\{(f, f) \mid f \leq f_A^*\} \cup \{(f_A^*, f_B) \mid f_A^* \leq f_B \leq m\}$ *for* $m = \max\left\{f_A^*, \sup\{f \mid c(f) = 0\}\right\}$*.*
3. *If* $g(0) > c'(0)$ *and* $g(f_A^*) < c'(f_A^*)$*, then* $\exists! f_B^*$ *satisfying* $0 < f_B^* < f_A^*$ *and* $g(f_B^*) = c'(f_B^*)$*, and the set of Nash equilibria is* $\{(f, f) \mid f \leq f_B^*\}$*.*

Proof. Note that

$$\frac{d}{df_A}\big(-g(f_A) + c'(f_A)\big) = -g'(f_A) + c''(f_A) > 0 \ \forall f_A \tag{50}$$

by (43) and $c''(f_A) \geq 0$.

For the first case, assume $g(0) \leq c'(0)$. Then $-g(f_A) + c'(f_A) > -g(0) + c'(0) \geq 0$ for any $f_A > 0$ (in particular, $-g(f_A^*) + c'(f_A^*) > 0$, implying that the first and second cases can never happen concurrently); and $c(f_A) > 0$ for any $f_A > 0$ since $c'(0) \geq g(0) > 0$. Thus, applying Lemma 1, at $f_A = 0$ the third case of (47) will be true, and at $f_A > 0$ the second case of (47) will be true. Then by definition, a Nash equilibrium $(\hat{f}_A, \hat{f}_B)$ satisfies $\hat{f}_A \in \arg\min_{0 \leq f_A \leq 1} C_A(f_A, \hat{f}_B)$ and $\hat{f}_B \in \arg\min_{0 \leq f_B \leq 1} C_A(\hat{f}_A, f_B)$, which we see can only be true for $(\hat{f}_A, \hat{f}_B) = (0, 0)$, since for any $\hat{f}_A > 0$ we have everything in $\arg\min_{0 \leq f_B \leq 1} C_A(\hat{f}_A, f_B)$ is $< \hat{f}_A$ while everything in $\arg\min_{0 \leq f_A \leq 1} C_A(f_A, \hat{f}_B)$ is $\geq \hat{f}_B$.

For the second case, we have $g(f_A^*) \geq c'(f_A^*)$, so $-g(f_A) + c'(f_A) \leq -g(f_A^*) + c'(f_A^*) \leq 0$ for any $f_A \leq f_A^*$. Then, applying Lemma 1, we see that $\{(f, f) \mid f \leq f_A^*\}$ are all Nash equilibria, and if $c(f_A^*) > 0$ then this is all of the Nash equilibria. However, if $c(f_A^*) = 0$, then the set of $\{(f_A^*, f_B) \mid f_A^* \leq f_B \leq m\}$ with $m = \sup\{f \mid c(f) = 0\}$ will also all be Nash equilibria by the third case of (47).

For the third case, we have $-g(0) + c'(0) < 0$ and $-g(f_A^*) + c'(f_A^*) > 0$, so by (50) there must be exactly one f_B^* strictly between 0 and f_A^* satisfying $g(f_B^*) = c'(f_B^*)$, with $g(f_A) \geq c'(f_A)$ for all $f_A \leq f_B^*$ and $g(f_A) < c'(f_A)$ for all $f_A > f_B^*$. Then, applying Lemma 1, we see that the set of Nash equilibria will be $\{(f, f) \mid f \leq f_B^*\}$. □

Corollary 1. *All Nash equilibria will have Country A sharing less resource than is optimal for it if and only if* $g(f_A^*) < c'(f_A^*)$.

Proof. If $g(f_A^*) < c'(f_A^*)$, then either the first or third case of Theorem 1 is true, and in either case all Nash equilibria will have Country A sharing less than its optimal f_A^*. And if $g(f_A^*) \geq c'(f_A^*)$, then the second case is true and any of the Nash equilibria in $\{(f_A^*, f_B) \mid f_A^* \leq f_B \leq m\}$ will have Country A sharing its optimal fraction f_A^*. □

Note that in the case of a linear distribution cost function $c(f_B) = \alpha f_B$, the criterion in Corollary 1 can be simplified to $g(f_A^*) < \alpha$. Applying this theory to our vaccine sharing simulations, we can interpret the criterion $g(f_A^*) < \alpha$ as saying "the marginal cost increase for Country B to reject the first vaccine at Country A's vaccine sharing optimum is less than Country B's cost of distributing one vaccine". If this statement is correct, then Country A will share less vaccines than its optimum at Nash equilibrium. This can be understood intuitively: if at Country A's optimum, Country B loses less epidemiologically from rejecting vaccines than it gains by saving distribution costs, then Country B will choose to reject vaccines and so Country A will share less than its optimum in order to avoid this scenario.

We show in Fig. B what $g(f_A)$ looks like in this system, that the assumption (43) is satisfied, and how the criterion of $g(f_A^*) < \alpha$ applies to our other game-theoretic results concerning distribution costs in Country B (main text Fig. 6 and Fig. J). And in Fig. C we show that the assumption (42) is also satisfied in our system.

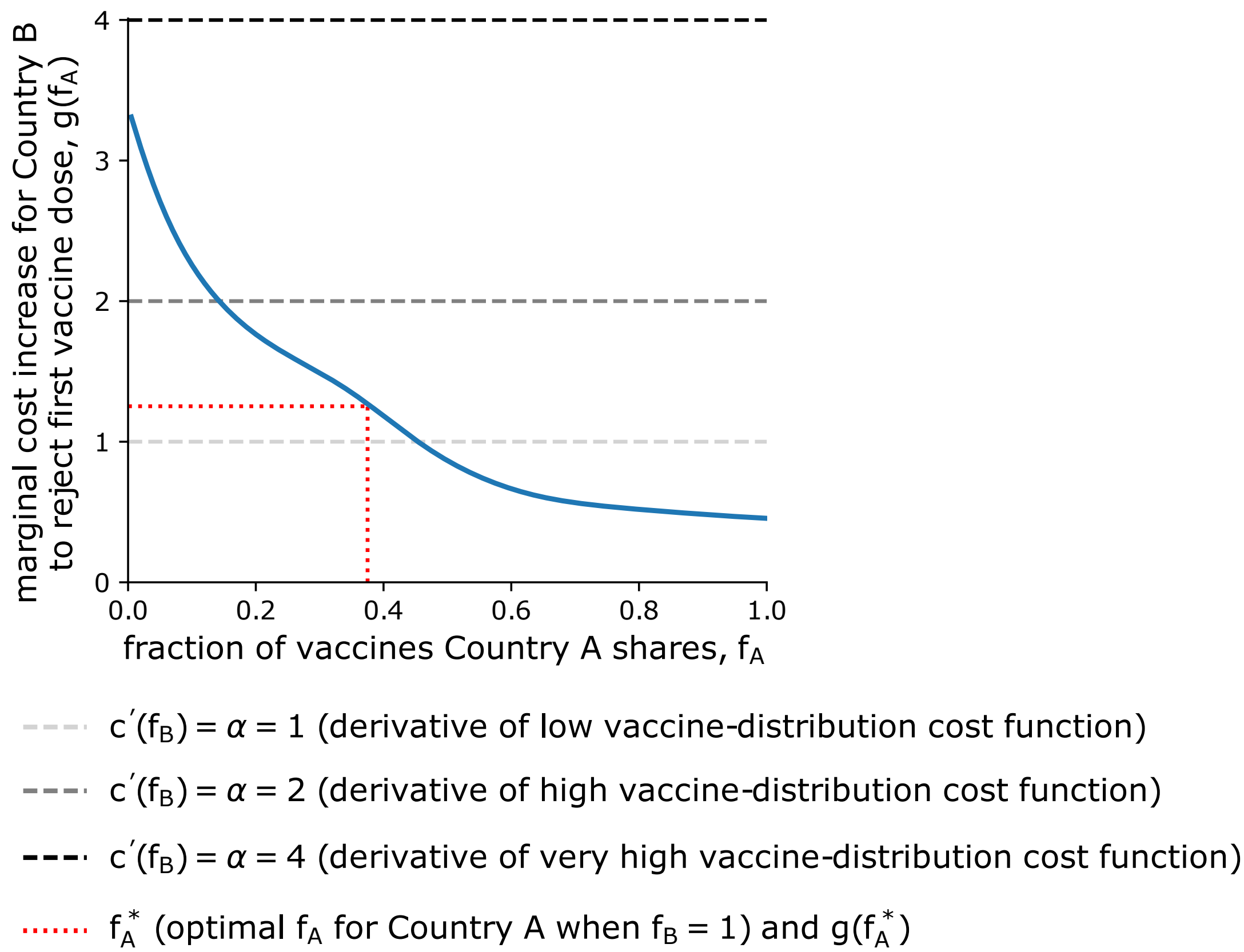

Figure B: Blue curve shows $g(f_A)$, the marginal cost increase to Country B to reject one vaccine, from our simulated vaccine sharing model. This curve decreases with f_A, thus satisfying (43) The red dotted lines show Country A's optimum vaccine sharing f_A^* and its value $g(f_A^*)$ under the function g. Other horizontal dashed lines show the values of α (the cost of distributing one vaccine relative to the cost of one infection) we explore in our other results: $\alpha = 1$ (light gray) and $\alpha = 2$ (dark gray) corresponding to the "low distribution cost" and "high distribution cost" scenarios in main text Fig. 6, respectively, and $\alpha = 4$ (black) and $\alpha = 0$ (not shown, but would overlap with the x-axis) correspond to the "very high distribution cost" and "very low distribution cost" scenarios in Fig. J. By Corollary 1, the α for which $g(f_A^*) < \alpha$, namely $\alpha = 2$ or 4 here, correspond to the scenarios where Country A will share less vaccines than its optimum at Nash equilibria; we see this to be case in main text Fig. 6 and Fig. J. Furthermore, we can calculate f_B^*, the maximum vaccine sharing fraction at Nash equilibrium (defined in Theorem 1), as $g^{-1}(\alpha)$ for any given α satisfying $g(1) < \alpha < g(0)$ (which here only applies to $\alpha = 0$ and 1).

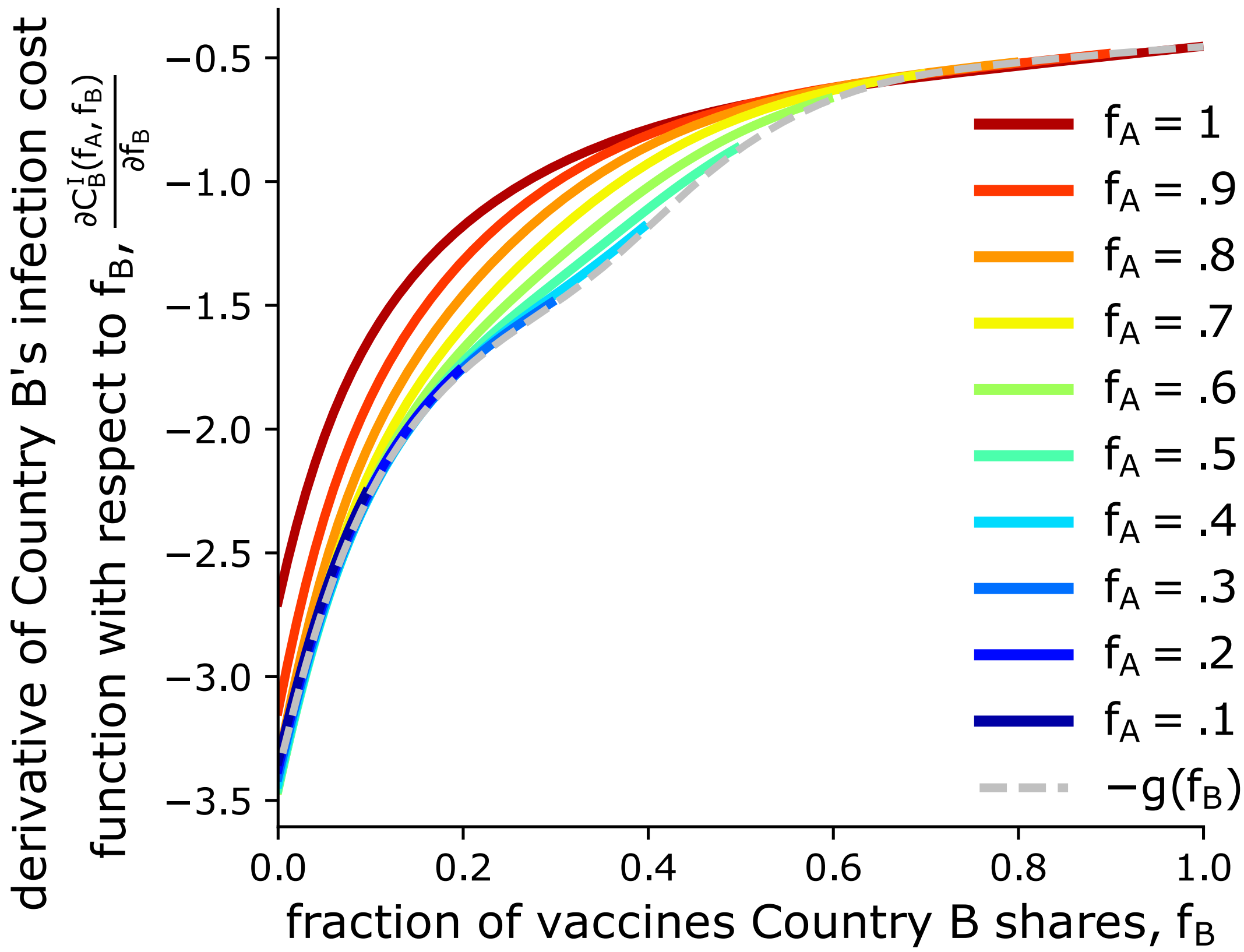

Figure C: Derivative of Country B's infection cost function with respect to f_B, $\frac{\partial C_B^I(f_A,f_B)}{\partial f_B}$, for different values of f_A and just over $f_B < f_A$. Each curve's color denotes its corresponding value of f_A. These curves are all increasing, thus satisfying (42). The gray curve shows $-g(f_B) = \frac{\partial^-}{\partial f_B} C_B^I(f_A, f_B)\Big|_{f_B=f_A}$ (a vertically flipped version of the blue curve in Fig. B), which connects all of the right endpoints of the colored curves here by definition and is increasing by (43).

3 Supplementary figures and results

3.1 Example with both country's dynamics and susceptibilities

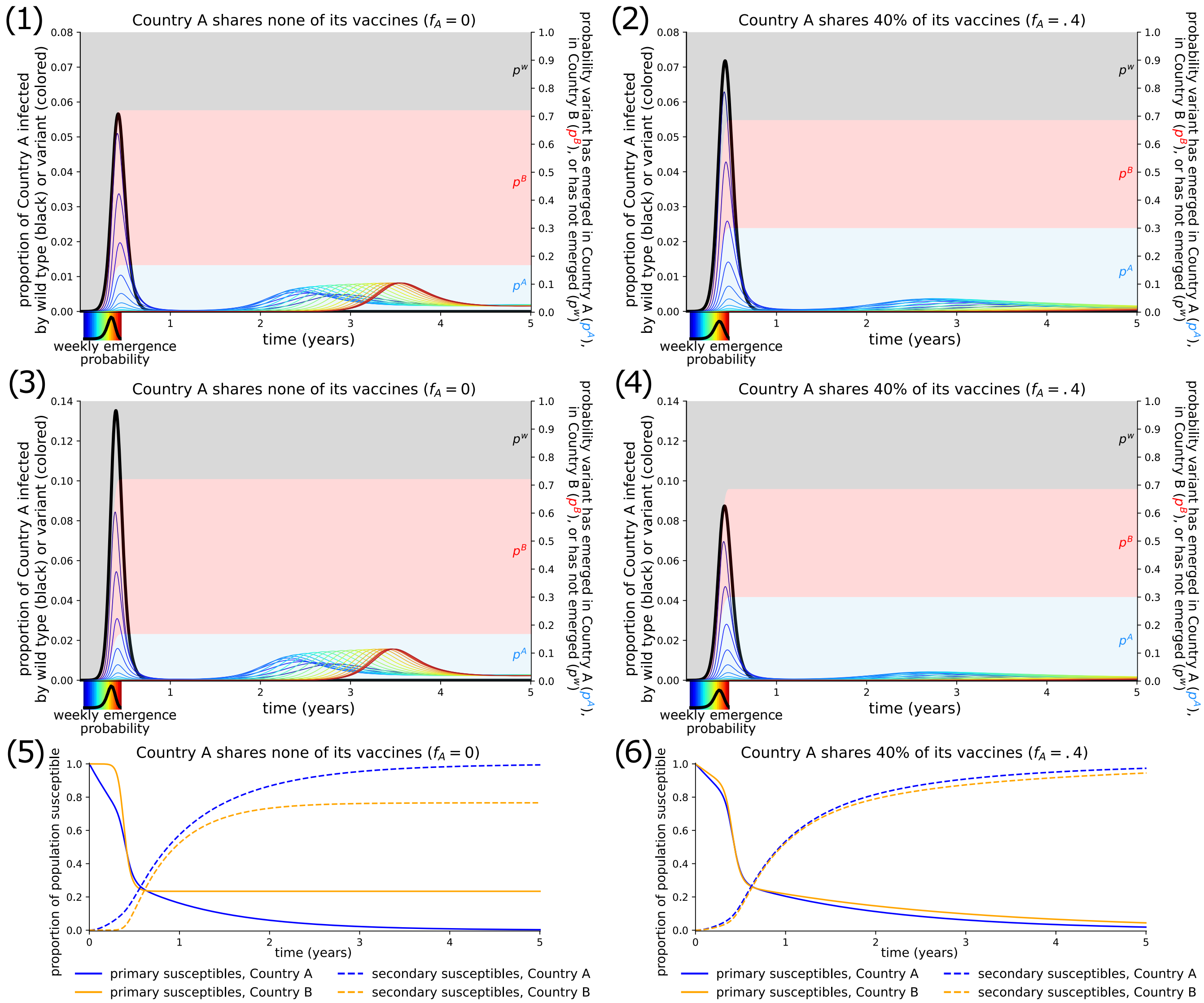

Figure D: Expanding on Fig. 2 in the main text. (1)–(4) Wild-type (black) and variant (colors) prevalence in Country A (top row) and Country B (middle row). Each variant prevalence curve represents one of many parallel scenarios, stratified and colored by the week of variant emergence. Color bars map each color to its corresponding variant emergence week on the x-axis above it, with the curves inside showing the probability of a variant emerging in each week. Background area plots show the probabilities a variant has emerged in Country A (p^A, blue), has emerged in Country B (p^B, pink), or has not yet emerged (p^w, gray). (5)–(6) show the proportion of the population susceptible primarily (solid) or secondarily (dashed) in Country A (blue) and Country B (orange). In the left column Country A is hoarding all vaccines, and in the right column Country A shares 40% of vaccines with Country B. Here, wild-type $R_0 = 2$, $\nu = 1$, $\mu = 5$, $\eta = .1$, and variant has a 10% transmission increase.

3.2 Example with low wild-type $R_0 = 1.5$ (vaccine sharing has evolutionary benefit instead of epidemiological benefit)

In the next two Figs. E–F, we explore the scenario that the wild-type R_0 is 1.5, instead of $R_0 = 2$ as we assume in most other figures in the main text in supplement. As we show in the main text, lower wild-type R_0 corresponds to a regime where vaccine sharing has an evolutionary benefit rather than an epidemiological benefit; in other words, vaccine sharing reduces the probability of the variant emerging at all, though it does not appreciably reduce variant infections given that it emerges. The effect is still that vaccine sharing provides tangible benefits to both countries, even if the means by which it does so is different than when R_0 is higher (for which the benefit of vaccine sharing is epidemiological, just reducing variant infections given emergence).

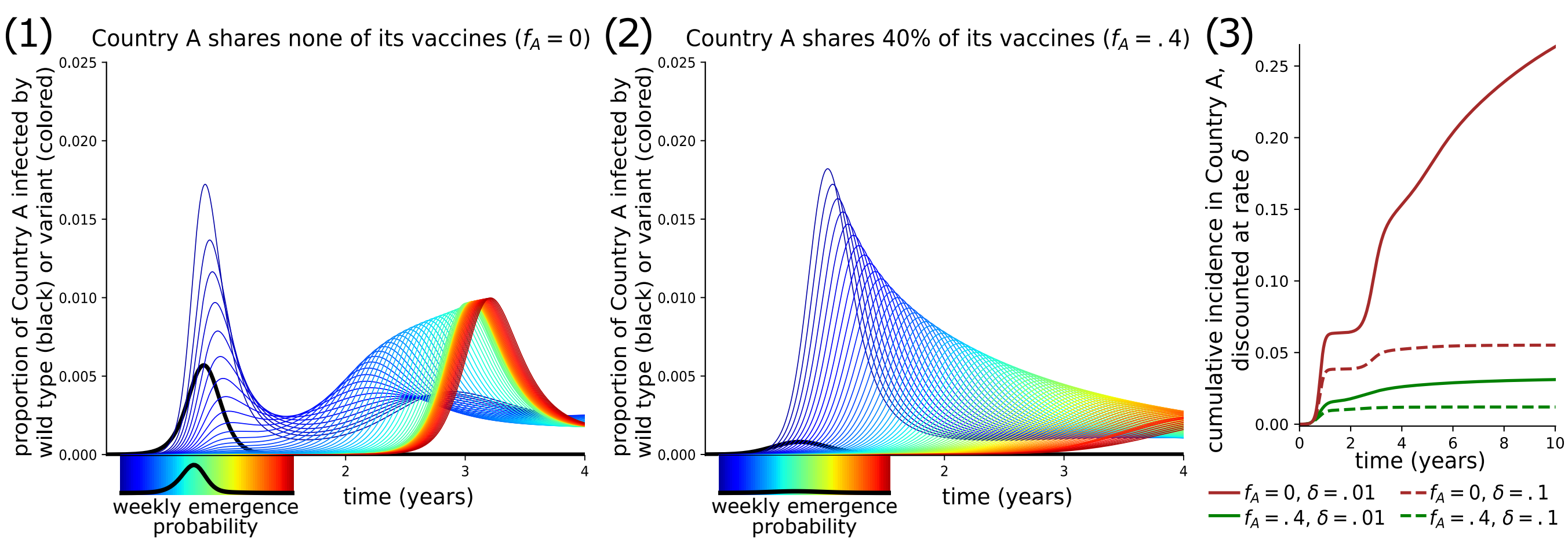

Figure E: Replica of Fig. 2 in the main text, except with the wild-type $R_0 = 1.5$ (instead of $R_0 = 2$). (1)–(2) Wild-type (black) and variant (colors) prevalence in Country A, which either (1) hoards all vaccines or (2) shares 40% of vaccines with Country B. Each variant prevalence curve represents one of many parallel scenarios, stratified and colored by the week of variant emergence. Color bars map each color to its corresponding variant emergence week on the x-axis above it, with the curves inside showing the probability of a variant emerging in each week. (3) Country A's cumulative infection cost, with infections discounted at annual rate δ of 1% (solid) or 10% (dashed), and with Country A either hoarding all vaccines (brown) or sharing 40% of its vaccines (green). The infection cost accounts for both wild-type infections and a weighted average of variant infections across all possible variant emergence scenario. Here, wild-type $R_0 = 1.5$, $\nu = 1$, $\mu = 5$, $\eta = .1$, and variant has a 10% transmission increase.

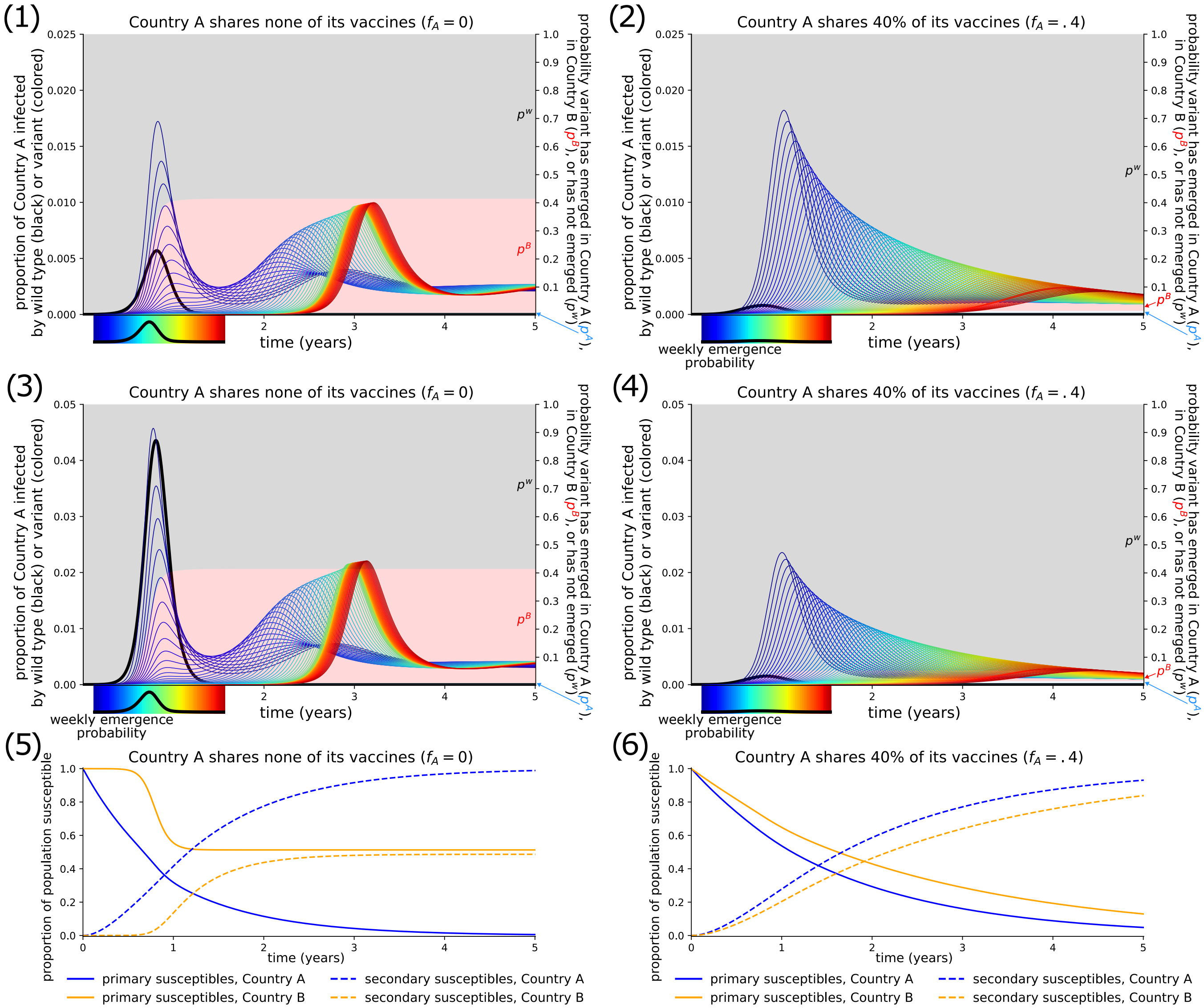

Figure F: Replica of Fig. D, except with the wild-type $R_0 = 1.5$ (instead of $R_0 = 2$). (1)–(4) Wild-type (black) and variant (colors) prevalence in Country A (top row) and Country B (middle row). Each variant prevalence curve represents one of many parallel scenarios, stratified and colored by the week of variant emergence. Color bars map each color to its corresponding variant emergence week on the x-axis above it, with the curves inside showing the probability of a variant emerging in each week. Background area plots show the probabilities a variant has emerged in Country A (p^A, blue), has emerged in Country B (p^B, pink), or has not yet emerged (p^w, gray). (5)–(6) show the proportion of the population susceptible primarily (solid) or secondarily (dashed) in Country A (blue) and Country B (orange). In the left column Country A is hoarding all vaccines, and in the right column Country A shares 40% of vaccines with Country B. Here, wild-type $R_0 = 1.5$, $\nu = 1$, $\mu = 5$, $\eta = .1$, and variant has a 10% transmission increase.

3.3 Variant emergence probabilities by country and mutation rate

Here we expand on main text Figs. 4B and 4D to break down variant emergence probability into probabilities of variant emergence in Country A and emergence in Country B (Fig. G). Total variant emergence probability ($p^A + p^B$) is a symmetric function of f_A with a minimum at $f_A = .5$. Breaking this function into probabilities of variant emergence in Country A (p^A) and emergence in Country B (p^B), we see it is the sum of an increasing function in f_A (p^A) and a decreasing function in f_A (p^B). We then show that this pattern holds under different mutation rates (Fig. H).

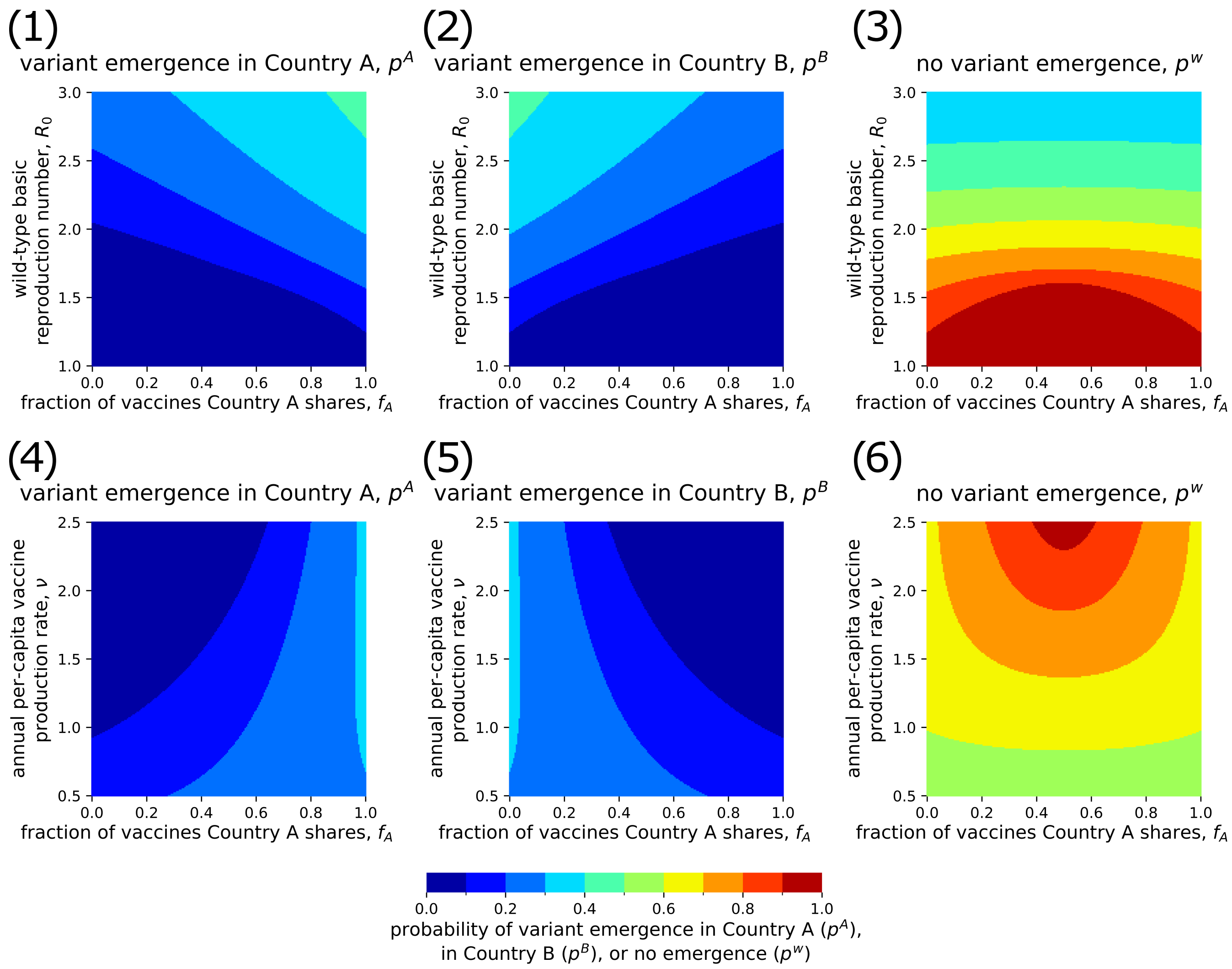

Figure G: Probability of a variant emerges in Country A (p^A, left column), emerges in Country B (p^B, middle column), or never emerges (p^w, right column), for varying wild-type R_0 (top row) or varying annual per-capita vaccine production rate ν (bottom row). The fraction f_A of vaccines Country A shares is also varied in all plots. Note $p^A + p^B + p^w = 1$. (3) and (6) are replicas of main text Figs. 4B and 4D, respectively, except reflected along the diagonal, subtracted from 1, and with a different color scale.

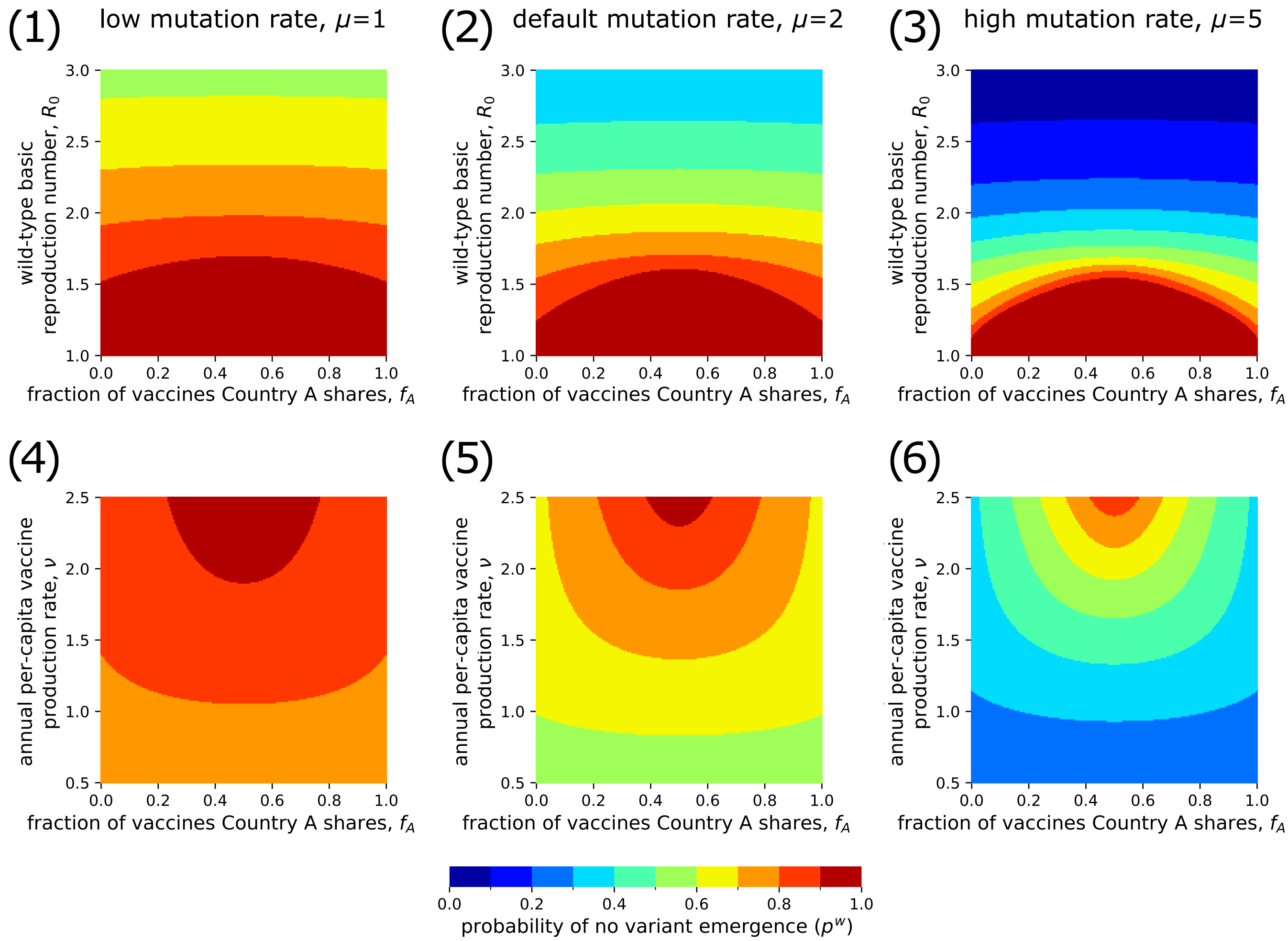

Figure H: Probability that no variant emerges (p^w, right column) for varying wild-type R_0 (top row) or varying annual per-capita vaccine production rate ν (bottom row). The fraction f_A of vaccines Country A shares is also varied in all plots. In the middle column, the default value of population-scaled mutation rate $\mu = 2$ is used, replicating Figs. G3 and G6, respectively. In the left column, a low mutation rate $\mu = 1$ is used, and in the right column, a high mutation rate $\mu = 5$ is used. For all mutation rates, the pattern of vaccine sharing having a greater evolutionary benefit for lower wild-type R_0 and higher vaccine production rate ν is maintained.

3.4 Variant has 10% transmission decrease (instead of 10% increase)

In the next figure, we explore scenarios in which the variant has a 10% transmission decrease from the wild-type, as opposed to a 10% transmission increase as we explore in all other figures in the main text in supplement. We see that variant infections can still remain a threat that warrants vaccine sharing as an optimal strategy, even when the variant is weaker than the wild-type by transmission rate. However, since the variant has a degree of immune escape, the advantage this brings can compensate the lower transmission rate and make it still a considerable future threat to be best addressed with some degree of vaccine sharing.

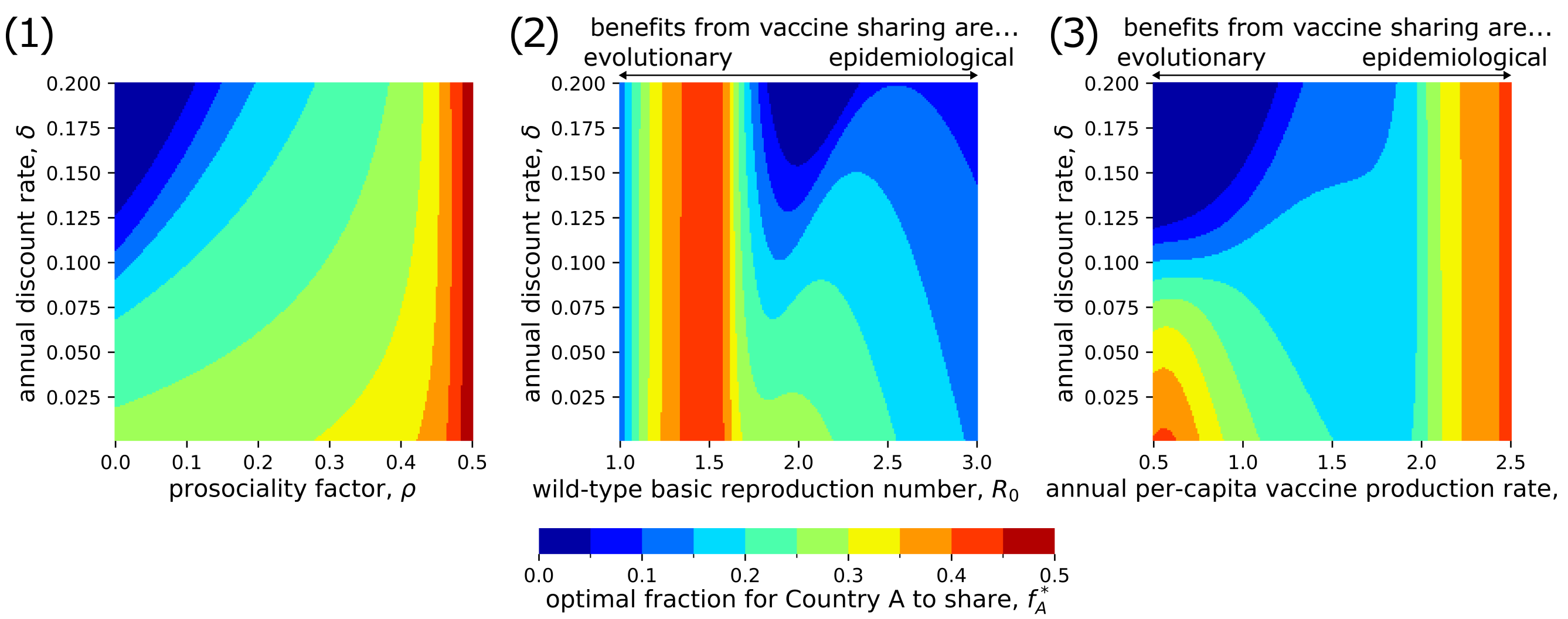

Figure I: Effects of varying (1) prosociality factor ρ, (2) wild-type R_0, and (3) annual per-capita vaccine production rate ν, along with annual discount rate δ, in scenarios with the variant having a 10% transmission decrease from the wild-type. (1)–(3) are replicas of main text Figs. 3C, 4A, and 4C, respectively, except with the variant having a 10% transmission decrease (instead of a 10% increase). In (2)–(3), benefits from vaccine sharing are either primarily evolutionary (reducing variant emergence probability) when wild-type R_0 is low or vaccine production is high, or primarily epidemiological (reducing variant cases given emergence) when wild-type R_0 is high or vaccine production is low.

3.5 More vaccine distribution cost functions $c(f_B)$

Here we expand the game-theoretic analysis of incorporating high vaccine distribution costs to Country B, as shown in Fig. 6 in the main text, to show results with either a very low vaccine distribution cost function ($c(f_B) = 0$) or a very high vaccine distribution cost function ($c(f_B) = 4f_B$). When distribution costs are very low Country B is able to always accept all of the vaccines shared with it, whereas when distribution costs are very high Country B is never able to accept any of the vaccines shared with it.

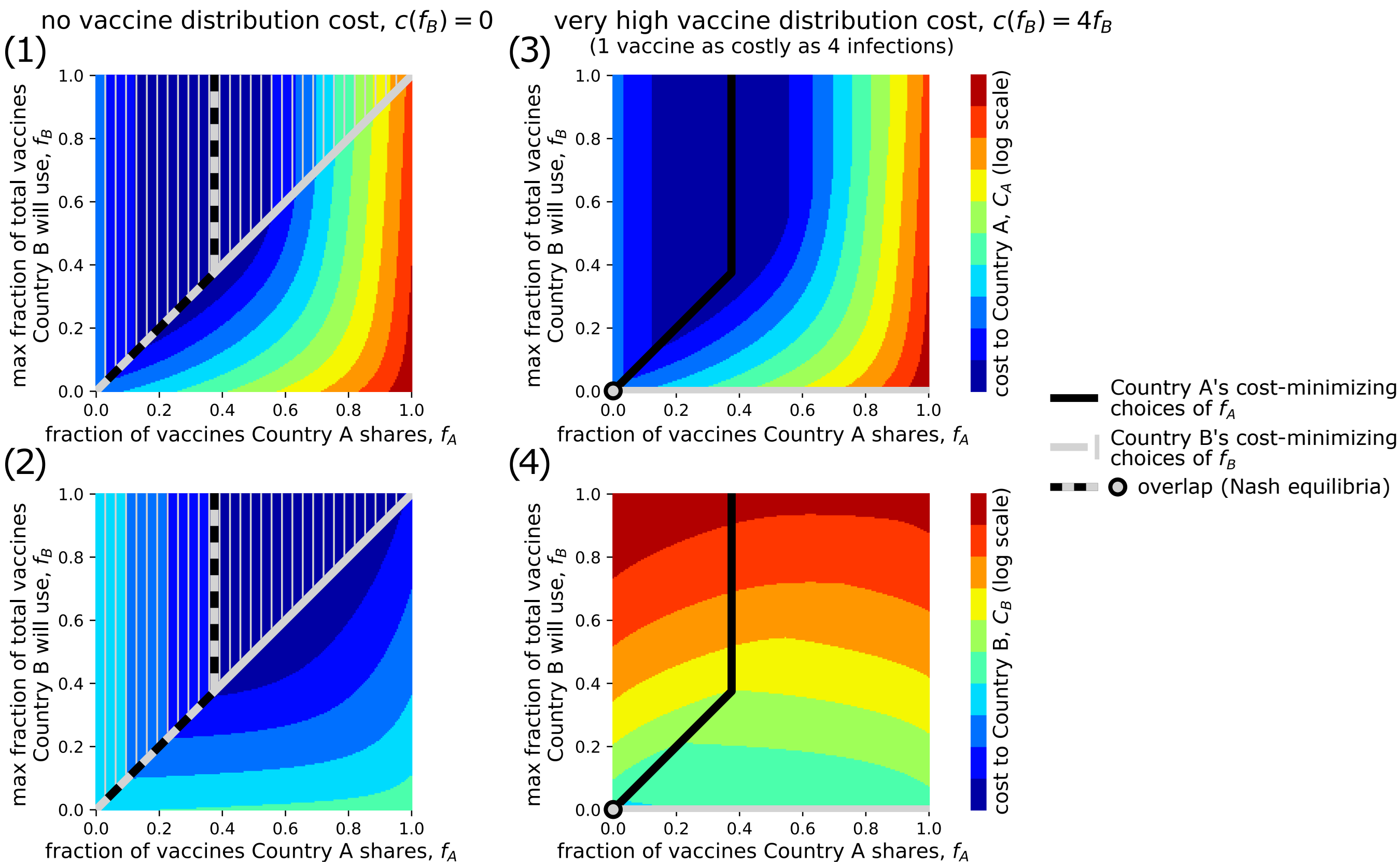

Figure J: Replica of main text Fig. 6A–D, except with vaccine distribution cost functions $c(f_B) = 0$ and $c(f_B) = 4f_B$. Infection costs to Country A (C_A, top row) and Country B (C_B, bottom row) with x-axes varying f_A (fraction of its vaccines that Country A shares with Country B) and y-axes varying f_B (the maximum fraction of vaccine production that Country B is able to accept), and with different vaccine distribution costs to Country B. In the left column, there is a very low vaccine distribution cost function $c(f_B) = 0$; and in the right column, there is a very high cost function $c(f_B) = 4f_B$, where one vaccine is as costly as four infections. Black curves show the values of f_A that minimize Country A's infection cost for each value of f_B, gray curves (or gray regions as in the left column) show the values of f_B that minimize Country B's infection cost for each value of f_A, and their overlaps (black-gray dashed line or dot) represent regions of Nash equilibria.

3.6 Country A helps with Country B's infrastructure costs

Here we address the possibility of Country A sharing the burden of Country B's high vaccine distribution costs, adding to the model that Country A can pay a fraction ζ of Country B's distribution cost $c(f_B)$. Furthermore, the costs may be less of a burden for Country A than for Country B (e.g. if Country A already has extra infrastructure, like cold chain storage, that they can donate to Country B); we allow for this possibility with a parameter x which describes the relative cost of developing vaccine distribution infrastructure for Country A relative to Country B. With these new additions, we update Eqs. (12)–(13) to

$$C_A = \sum_{k=1}^{K} e^{-\delta\tau_k} \left((1-\rho)J_A(\tau_k) + \rho J_B(\tau_k)\right) + x\zeta c(f_B) \tag{51}$$

$$C_B = \sum_{k=1}^{K} e^{-\delta\tau_k} \left((1-\rho)J_B(\tau_k) + \rho J_A(\tau_k)\right) + (1-\zeta)c(f_B). \tag{52}$$

In this analysis, we assume that $f_A = f_B$, or that Country B decides how much infrastructure they are willing to invest in with the knowledge that Country A will donate as much vaccines as Country B can possibly distribute. Then, with Country B choosing a value of f_B (which is also equal to f_A), Country A chooses a value of ζ. By Eq. (51), we see that Country A's cost-minimizing value of ζ will always be $\zeta = 0$ for any specific choice of f_B that Country B chooses. Thus, any Nash equilibrium will always have Country A not helping with Country B's distribution infrastructure costs. However, there may be choices of (ζ,f_B) where Country A does help with infrastructure costs ($\zeta > 0$) that are more optimal for both countries than the Nash equilibrium with $\zeta = 0$. We show this in Fig. K: there is a region of points in the ζ–f_B space where $\zeta > 0$ and where both countries have lower costs that at Nash equilibrium; and these regions are larger when distribution infrastructure is less costly for Country A (smaller x). Thus, helping with vaccine distribution costs may be optimal if both countries coordinate their response, but this is not possible at a Nash equilibrium where Country A assisting with distribution infrastructure costs is decided independently of Country B's investment into distribution infrastructure.

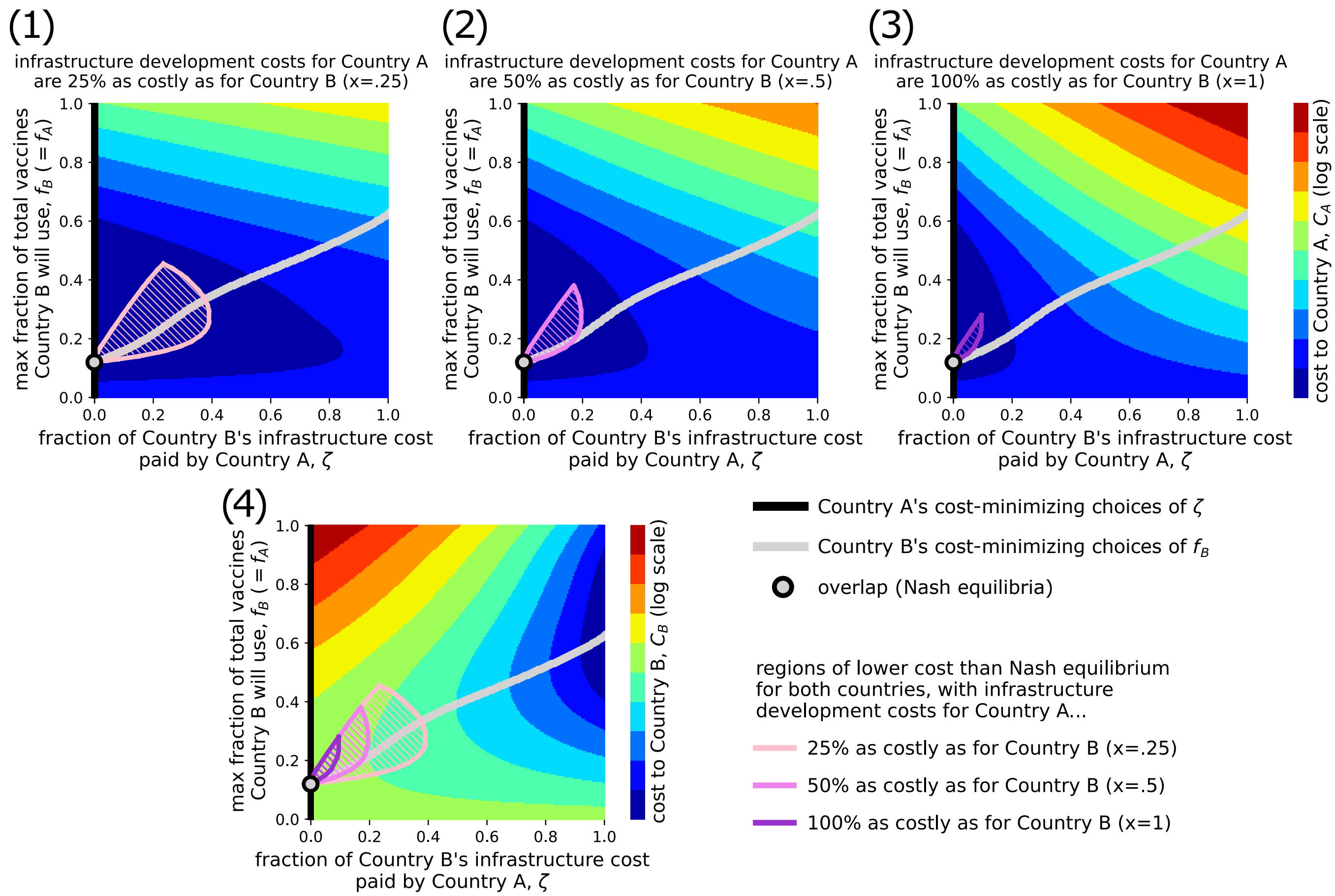

Figure K: Costs to Country A (1–3) and Country B (4), with Country B investing different amounts into its vaccine distribution infrastructure (determined by f_B, the maximum fraction of total vaccine production that Country B is able to accept) and Country A paying for different fractions ζ of Country B's infrastructure costs. We assume for these plots that $f_A = f_B$, that is Country B chooses its infrastructure investment and then Country A shares exactly the amount of vaccines that Country B can afford to distribute. We also vary x, the relative cost of paying for vaccine distribution infrastructure for Country A as compared to Country B; we either let (1) $x = .25$ (distribution infrastructure is only 25% as costly to Country A as Country B), (2) $x = .5$ (distribution infrastructure is 50% as costly to Country A as Country B), or (3) $x = 1$ (distribution infrastructure is equally costly for both countries). Black curves show Country A's cost-minimizing values of ζ for each of Country B's choice of f_B (the optimal choice for Country A is always $\zeta = 0$, corresponding to no assistance with Country B's distribution infrastructure); gray curves show Country B's cost-minimizing values of f_B for each of Country A's choice of ζ; and their point of overlap is the Nash equilibrium. None of the cost-minimizing lines or Nash equilibria changes with x. Colored shaded areas are regions in the ζ–f_B space where both countries sustain lower costs than at the Nash equilibrium, with different colors for different values of x.

References

[1] Saad-Roy, C. M. *et al.* Immune life history, vaccination, and the dynamics of SARS-CoV-2 over the next 5 years. *Science* **370**, 811–818 (2020). URL https://www.science.org/doi/full/10.1126/science.abd7343.

[2] Morris, S. E. *et al.* Demographic buffering: titrating the effects of birth rate and imperfect immunity on epidemic dynamics. *Journal of The Royal Society Interface* **12**, 20141245 (2015). URL https://royalsocietypublishing.org/doi/10.1098/rsif.2014.1245.

[3] Nielsen, B. F., Saad-Roy, C. M., Metcalf, C. J. E., Viboud, C. & Grenfell, B. T. Eco-evolutionary dynamics of pathogen immune-escape: deriving a population-level phylodynamic curve. *Journal of The Royal Society Interface* **22**, 20240675 (2025). URL https://royalsocietypublishing.org/doi/full/10.1098/rsif.2024.0675.

[4] Nash, J. Non-Cooperative Games. *Annals of Mathematics* **54**, 286–295 (1951). URL https://www.jstor.org/stable/1969529.
