# Supplementary material for: Economic factors promoting vaccine nationalism in the face of viral evolution
Source: PLoS Comput Biol. 2026 Aug 31;22(8):e1014466. doi: 10.1371/journal.pcbi.1014466 (PMC13541263; doi:10.1371/journal.pcbi.1014466)
Supplement: Supplementary file 2 [file pcbi.1014466.s002.pdf]

Report to the authors.

RE: *Economic factors promoting vaccine nationalism in the face of viral evolution*
Submitted to **PLOS Computational Biology**, PCOMPBIOL-D-25-02016.

1 Summary of paper.

The paper presents a theoretical investigation of the incentives a country has to hoard vaccines during a communicable disease epidemic. Motivated by the global distribution of vaccines during the early phases of the COVID-19 pandemic, a model is developed in which a vaccine-developing country may choose to share a vaccine with another country. The developing country benefits for altruistic reasons and to the extent that sharing vaccines early reduces the probability variants will evolve in the other country.

2 Critique.

This is a well-crafted paper which addresses issues of obvious policy importance. However, the contributions are minor: the major result is a rich country (country A) will be more likely to share vaccinations with a poor country (country B) if A is altruistic, or if A is more worried about variants evolving in B and returning to A. Since the latter occurs over time, country A will also be more willing to share more vaccines if it has a lower temporal discount rate. I think these points would also fall readily out of a very simple two-period model without a compartmental disease model grafted on top, and the full model if anything obscures the underlying economic and epidemiological intuition.

A highlighted result is that country A will not be able to share as many vaccines as it would like if the marginal epidemiological benefits in the poor country of expanding capacity are less than the marginal costs incurred by the poor country of expanding capacity (supplemental material Corollary 1). This result obtains simply because the poor country rejects vaccines for which it would cost more to distribute locally than B obtains in benefits from reduced infections, and is also a straightforward result which could be demonstrated in a much simpler setting. A related, also emphasized result is that country A will not subsidize B's vaccine infrastructure. These results are derived in the generic static game (which also excludes altruism) laid out in section 2 of the supplement rather than the main model itself.

But these results hinge on arbitrary modeling assumptions and lack solid economic intuition. First, country A exhibits only a limited form of altruistic preferences: it cares

about the epidemiological costs incurred in B, but does not care about infrastructure costs in B. Second, there is no mechanism in the model which allows countries to directly transfer resources other than vaccines. In a more general model in which A cares about B's overall well-being instead of only disease-induced mortality, country A would at least partially internalize both the epidemiological and the infrastructure costs incurred by B, and A then has both the incentive and the capability to make monetary transfers.

Generally, in the absence of other distortions, in this environment A could simply make a monetary transfer to B and an efficient outcome would obtain, since A knows B will then optimally allocate the transfer across vaccination and other welfare-enhancing expenditures. That country A has an incentive to induce B to produce more vaccine infrastructure than B would prefer privately could also be explored in this model.

Finally, the strategic interaction aspect of the model is only of any interest when country A would like to give country B more vaccines than country B is willing to accept, but the entire point of the paper, and the subject of this branch of the multiple intertwined literatures, is that richer countries hoarded the vaccine supply during COVID-19, not that they were thwarted in their attempts to give vaccines to other countries by inadequate infrastructure.
