# Supplementary material for: Economic factors promoting vaccine nationalism in the face of viral evolution
Source: PLoS Comput Biol. 2026 Aug 31;22(8):e1014466. doi: 10.1371/journal.pcbi.1014466 (PMC13541263; doi:10.1371/journal.pcbi.1014466)
Supplement: Supplementary file 3 [file pcbi.1014466.s003.pdf]

Reviewer 1

This paper innovates the application of epi-evolutionary-economic modelling to vaccine nationalism. The paper is conceptually novel, and vaccine nationalism is a pressing public health problem that is likely to emerge again in the future. The authors develop a 2-country model of viral transmission, vaccine and infection costs, and mutation to determine when countries that can manufacture vaccines are incentivized to share vaccines with countries that cannot. The analysis shows, among other things, that temporal discounting, prosociality (interpreted in this case as preferring one's own country over another) and infrastructure costs are important drivers. The paper is interesting to read and well-written, so I only have a few minor suggestions for revisions:

We are glad you enjoyed the paper. Thank you very much for your kind words and thoughtful comments.

1. The selection of parameter values was not always systematic or well-motivated. This is not much of an issue for an exploratory analysis such as this one, but it still might be helpful to see a table of parameter definitions, baseline parameter values (and/or ranges) and references. The parameter values seem in the right empirical ballpark, with a few exceptions (below, #3).

This is a great point. We have already included 2 tables with parameter values in the Supplement (Tables 1-2). We have it in the supplement now to avoid overcrowding the main text, and because many of the variables/parameters mentioned in these tables are only mentioned in the supplement. The idea to add ranges for the parameter values we explore in addition to their default values is a good one, and we now have added a column for that in Supplement Tables 1-2.

2. The paper already has a good section on Caveats to highlight model limitations. I would suggest only two more limitations to add to this section:
a. The model assumes that the vaccine blocks transmission, which may not always be the case, especially for MRNA vaccines.
b. The model assumes only two countries. However, in a multi-country model, I can see how vaccine-sharing might never be a Nash equilibrium. In a situation where all vaccine-producing countries are sharing their vaccines, an individual vaccine-produced country will be tempted to defect by hoarding their vaccines, thereby obtaining both the benefits of hoarding and the benefits of reduced immune-escape variants thanks to the sharing of the other vaccine-producing countries.

Thank you for bringing up these excellent points. We have now noted the first of these assumptions in lines 401-402 of the Caveats section, and the second of these in a new paragraph (lines 376-383) near the end of the Discussion section, since we believe bringing up the possibility of studying free-riding between multiple producing countries is a very salient avenue for future research.

3. Figure 6: I had a hard time deciding whether either c(f_B)= f_B or c(f_B) = 2f_B are fair assumptions. It might be for a low CFR pandemic like COVID-19, but for a higher CFR pandemic such as might be caused by HPAI, it might not be the case that per capita vaccine costs are the same order of magnitude as per capita infection costs.

This is a great point. In the supplement, we also plot a scenario where c(f_B) = 0 (Fig. S10), but this just reduces to the original model where Country B is assumed to accept all vaccines, since there would be no monetary incentive for Country B to reject any vaccines. We suspect that for a high CFR pandemic like HPAI, with 0 < c(f_B) / f_B << 1, this would behave qualitatively very similar to the case where c(f_B) = 0 and thus the case where Country B is not limited by infrastructure costs.

We have now added a sentence in the main text (lines 336-340) making these points.

4. Discussion: As a suggestion, the authors may wish to mention parallel literature on modelling climate change decision-making. Both are challenges to the Global Commons that require coordination, but they have very different mechanisms.

We thank you for this excellent idea. This is something we have also thought about, and including a small introduction to this important parallel fits very well at the end of our Discussion, where we have now added a new paragraph on this topic (lines 384-392).

5. Acronyms are introduced in a few places without being first defined, such as Gavi (should be all caps, page 4) and HPAI (page 22).

We appreciate you pointing out our lapse of defining the acronym for GAVI. We now do this on line 58. We had already defined HPAI on line 56, but thank you for considering this.

6. Page 4: I think “incentivized away” should “disincentivized”, although it is a nice turn of phrase.

Thank you for this suggestion, we have changed this now (line 71), as simpler is better.

<u>Reviewer 2</u>

The paper presents a theoretical investigation of the incentives a country has to hoard vaccines during a communicable disease epidemic. Motivated by the global distribution of vaccines during the early phases of the COVID-19 pandemic, a model is developed in which a vaccine-developing country may choose to share a vaccine with another country. The developing country benefits for altruistic reasons and to the extent that sharing vaccines early reduces the probability variants will evolve in the other country.

This is a well-crafted paper which addresses issues of obvious policy importance. However, the contributions are minor: the major result is a rich country (country A) will be more likely to share

vaccinations with a poor country (country B) if A is altruistic, or if A is more worried about variants evolving in B and returning to A. Since the latter occurs over time, country A will also be more willing to share more vaccines if it has a lower temporal discount rate. I think these points would also fall readily out of a very simple two-period model without a compartmental disease model grafted on top, and the full model if anything obscures the underlying economic and epidemiological intuition.

Thank you for your kind words and for taking the time to read and understand the paper and supplement. It is perhaps true that one could replicate some of the basic results of our paper with a simpler, two-period model without consideration of the underlying disease processes. However, we believe a model which can properly represent the epidemiological and economic processes at play in a more realistic temporal setting is necessary here for several reasons:

- A two-period model with no consideration of disease transmission would require many arbitrary assumptions about how the disease state and variant evolution probabilities differ between the two time periods, with no clear notion of how you would link the two time periods together in a meaningful way. And having an objective function just summing up results at just two disjoint time points would be too simple for the desired purpose of this paper.
- Our more nuanced model not only shows that vaccine sharing is in the vaccine-producing country's interest, but does so quantitatively and shows how this changes with key epidemiological and economic parameters. While some of these trends are more intuitive (e.g. lower discounting and higher altruism promote greater vaccine sharing), others are more surprising. Most notably, we highlight the unintuitive result that vaccine sharing is effective at lower R0 to reduce the probability of variant emergence, vaccine sharing is effective at higher R0 to reduce the number of infections given a variant does emerge, but at intermediate R0 vaccine sharing is much less effective (Fig. 4A-B). Similarly, our model allows us to show that vaccine sharing efficacy can be maximized at intermediate values of the viral mutation rate (Fig. 5B), i.e. vaccine sharing may not be incentivized for a virus mutating too slowly for evolution to be a concern or too quickly for policy targeting variant evolution to be effective.
- These examples are especially only possible to show using the novel methodology we employ to link epidemiology and variant evolution probability over continuous time. We believe that this novel framework has merit on its own for the important problem it solves in the world of epi modeling: calculating the probability of variant evolution over continuous time in a principled manner. This method then allows us to disentangle how vaccine sharing efficacy is driven by reducing variant evolution probability vs reducing the number of infections given variant evolution.

While our model may seem complicated at first, we believe it is the simplest model that can capture all of the objectives we have mentioned here in a principled manner. This is not to discount the value a simpler model could have, especially in generalizing beyond epidemiology, but this would have precluded us from investigating the epidemiological problems we address in the nuance we have presented.

We have added a few sentences in lines 112-114 and 437-442 to clarify these points.

A highlighted result is that country A will not be able to share as many vaccines as it would like if the marginal epidemiological benefits in the poor country of expanding capacity are less than the marginal costs incurred by the poor country of expanding capacity (supplemental material Corollary 1). This result obtains simply because the poor country rejects vaccines for which it would cost more to distribute locally than B obtains in benefits from reduced infections, and is also a straightforward result which could be demonstrated in a much simpler setting. A related, also emphasized result is that country A will not subsidize B's vaccine infrastructure. These results are derived in the generic static game (which also excludes altruism) laid out in section 2 of the supplement rather than the main model itself.

Thank you for pointing out that this result, that country A shares less vaccines when country B's distribution capacity is limited, would fall from a simpler, more general model. This is precisely what we are showing in Section 2 of the supplement. This static game we present there is a generalization of the game we present in the main text. In particular, one can plug in the expressions C_A and C_B defined by the supplement Eqs. 6-9 into the functions $C_A^I(f_A,f_B)$ and $C_B^I(f_A,f_B)$ that form the basis of the simple game theoretic model in supplement Section 2. Doing so satisfies all of the assumptions about $C_A^I(f_A,f_B)$ and $C_B^I(f_A,f_B)$ laid out in Eqs. 37-43 (most of these follow from common sense about how our vaccine sharing model is set up, but we show numerically that the assumptions in Eq. 42-43 hold in Figs. S2-3). We've now made this connection more clear in the new sentence after Eq. 43.

By proving more general results about resource sharing when the receiving party has limited distribution capacity, as we do in supplement Section 2, we provide analytic support to the trends we show numerically in the main text Section 2.4 and Fig. 6 (this is mentioned in the main text paragraph lines 341-350).

But these results hinge on arbitrary modeling assumptions and lack solid economic intuition. First, country A exhibits only a limited form of altruistic preferences: it cares about the epidemiological costs incurred in B, but does not care about infrastructure costs in B. Second, there is no mechanism in the model which allows countries to directly transfer resources other than vaccines. In a more general model in which A cares about B's overall well-being instead of only disease-induced mortality, country A would at least partially internalize both the epidemiological and the infrastructure costs incurred by B, and A then has both the incentive and the capability to make monetary transfers.

Generally, in the absence of other distortions, in this environment A could simply make a monetary transfer to B and an efficient outcome would obtain, since A knows B will then optimally allocate the transfer across vaccination and other welfare-enhancing expenditures. That country A has an incentive to induce B to produce more vaccine infrastructure than B would prefer privately could also be explored in this model.

This is an excellent point, which we believe we have already addressed in an analysis in the supplement (Section 3.6, “Country A helps with Country B’s infrastructure costs”). We’ve put this analysis in the supplement to avoid overcomplicating the main text and we felt it a less central result to the focus of this paper. We point to this supplementary analysis in the main text in the paragraph in lines 360-375. We’ve added a new first sentence to this paragraph (lines 360-363) to make it clearer that we are addressing the possibility of Country A helping with Country B’s infrastructure costs and the potential benefit this can have.

Finally, the strategic interaction aspect of the model is only of any interest when country A would like to give country B more vaccines than country B is willing to accept, but the entire point of the paper, and the subject of this branch of the multiple intertwined literatures, is that richer countries hoarded the vaccine supply during COVID-19, not that they were thwarted in their attempts to give vaccines to other countries by inadequate infrastructure.

We appreciate you pointing out this important nuance in the language we use. You are correct that the word “hoarding” may hold the connotation that Country A is only stockpiling vaccines due to selfish reasons, not due a lack of trying. We have been using “hoarding” in a more general sense to refer to any observed stockpiling of vaccines beyond what is needed for the country’s populace. We have now attempted to use more clear language without the word “hoarding” when referring to Country A keeping vaccines because Country B cannot accept them all (lines 79-82 and 358-359).
