# Supplementary material for: Economic factors promoting vaccine nationalism in the face of viral evolution
Source: PLoS Comput Biol. 2026 Aug 31;22(8):e1014466. doi: 10.1371/journal.pcbi.1014466 (PMC13541263; doi:10.1371/journal.pcbi.1014466)
Supplement: Supplementary file 4 [file pcbi.1014466.s004.pdf]

We are very thankful to the AE for seeing the merit in our work and allowing a compromise from the reviewer's continued concerns. We have addressed these concerns in our latest draft:

"The authors should shorten or de-emphasise the section about the effects of pro-sociality on model results. And they should add some specific caveats to section 3 acknowledging that pro-sociality is modelled in a narrow and highly simplified way…"

> We have removed panel B from Fig. 3, which was focused on the effect of prosociality alone and was not necessary to the paper. We greatly shortened the 4th paragraph of Section 2.1, which focused on prosociality, and put it at the end of the previous paragraph. We also shortened the discussion on prosociality in the following paragraph (now the 4th paragraph of Section 2.1). Finally, we increased our discussion of the caveats of how we modeled prosociality, both in the last paragraph of Section 2.1 and in the 4th paragraph of the caveats Section 3. We hope these changes to de-emphasize the results of prosociality and further emphasize the caveats on how we model it are sufficient.

"...and the possibility that more efficient outcomes could be obtained by cash transfers rather than vaccine donations."

We have added a sentence in the caveats Section 3 at the end of the 4th paragraph addressing this possibility.
