# Supplementary material for: Economic factors promoting vaccine nationalism in the face of viral evolution
Source: PLoS Comput Biol. 2026 Aug 31;22(8):e1014466. doi: 10.1371/journal.pcbi.1014466 (PMC13541263; doi:10.1371/journal.pcbi.1014466)
Supplement: Peer review history

# Peer review: Decision Letter 0

**DOI:** 10.1371/journal.pcbi.1014466.r001

**Authors:** Roger Dimitri Kouyos, Michael J Plank

25 Dec 2025

Economic factors promoting vaccine nationalism in the face of viral evolution

PLOS Computational Biology

Dear Dr. Freedman,

Thank you for submitting your manuscript to PLOS Computational Biology. After careful consideration, we feel that it has merit but does not fully meet PLOS Computational Biology's publication criteria as it currently stands. Therefore, we invite you to submit a revised version of the manuscript that addresses the points raised during the review process.

Please submit your revised manuscript by Feb 24 2026 11:59PM. If you will need more time than this to complete your revisions, please reply to this message or contact the journal office at ploscompbiol@plos.org. When you're ready to submit your revision, log on to https://www.editorialmanager.com/pcompbiol/ and select the 'Submissions Needing Revision' folder to locate your manuscript file.

Please include the following items when submitting your revised manuscript:

* A rebuttal letter that responds to each point raised by the editor and reviewer(s). You should upload this letter as a separate file labeled 'Response to Reviewers'. This file does not need to include responses to formatting updates and technical items listed in the 'Journal Requirements' section below.

* A marked-up copy of your manuscript that highlights changes made to the original version. You should upload this as a separate file labeled 'Revised Manuscript with Track Changes'.

* An unmarked version of your revised paper without tracked changes. You should upload this as a separate file labeled 'Manuscript'.

If you would like to make changes to your financial disclosure, competing interests statement, or data availability statement, please make these updates within the submission form at the time of resubmission. Guidelines for resubmitting your figure files are available below the reviewer comments at the end of this letter

We look forward to receiving your revised manuscript.

Kind regards,

Michael J Plank

Academic Editor

PLOS Computational Biology

Roger Kouyos

Section Editor

PLOS Computational Biology

**Additional Editor Comments:**

AE comments: The reviewers are split with one broadly supportive and one who feels that the results are not sufficiently novel to merit publication. Particularly important is the reviewer’s comment that the key results could be obtained with a simpler model, and that the SIRS model does not illuminate (or even obscures) the important driving factors. On balance, having read the article and reviewers’ comments, I think that the paper has potential to be publishable and therefore encourage the authors to resubmit a revised version. However, in order to be considered further, the authors will need to explain very clearly what value a dynamic epidemic model adds and why the results could not be obtained with a simpler model.

**Journal Requirements:**

1) We ask that a manuscript source file is provided at Revision. Please upload your manuscript file as a .doc, .docx, .rtf or .tex. If you are providing a .tex file, please upload it under the item type u2018LaTeX Source Fileu2019 and leave your .pdf version as the item type u2018Manuscriptu2019.

2) Please upload all main figures as separate Figure files in .tif or .eps format. For more information about how to convert and format your figure files please see our guidelines:

https://journals.plos.org/ploscompbiol/s/figures

3) We have noticed that you have uploaded Supporting Information files, but you have not included a list of legends. Please add a full list of legends for your Supporting Information files after the references list.

**Reviewers' comments:**

Reviewer's Responses to Questions

**Comments to the Authors:**

**Please note here if the review is uploaded as an attachment.**

Reviewer #1: This paper innovates the application of epi-evolutionary-economic modelling to vaccine nationalism. The paper is conceptually novel, and vaccine nationalism is a pressing public health problem that is likely to emerge again in the future. The authors develop a 2-country model of viral transmission, vaccine and infection costs, and mutation to determine when countries that can manufacture vaccines are incentivized to share vaccines with countries that cannot. The analysis shows, among other things, that temporal discounting, prosociality (interpreted in this case as preferring one’s own country over another) and infrastructure costs are important drivers. The paper is interesting to read and well-written, so I only have a few minor suggestions for revisions:

1. The selection of parameter values was not always systematic or well-motivated. This is not much of an issue for an exploratory analysis such as this one, but it still might be helpful to see a table of parameter definitions, baseline parameter values (and/or ranges) and references. The parameter values seem in the right empirical ballpark, with a few exceptions (below, #3).

2. The paper already has a good section on Caveats to highlight model limitations. I would suggest only two more limitations to add to this section:

a. The model assumes that the vaccine blocks transmission, which may not always be the case, especially for MRNA vaccines.

b. The model assumes only two countries. However, in a multi-country model, I can see how vaccine-sharing might never be a Nash equilibrium. In a situation where all vaccine-producing countries are sharing their vaccines, an individual vaccine-produced country will be tempted to defect by hoarding their vaccines, thereby obtaining both the benefits of hoarding and the benefits of reduced immune-escape variants thanks to the sharing of the other vaccine-producing countries.

3. Figure 6: I had a hard time deciding whether either c(f_B)= f_B or c(f_B) = 2f_B are fair assumptions. It might be for a low CFR pandemic like COVID-19, but for a higher CFR pandemic such as might be caused by HPAI, it might not be the case that per capita vaccine costs are the same order of magnitude as per capita infection costs.

4. Discussion: As a suggestion, the authors may wish to mention parallel literature on modelling climate change decision-making. Both are challenges to the Global Commons that require coordination, but they have very different mechanisms.

5. Acronyms are introduced in a few places without being first defined, such as Gavi (should be all caps, page 4) and HPAI (page 22).

6. Page 4: I think “incentivized away” should “disincentivized”, although it is a nice turn of phrase.

Reviewer #2: Attached.

**********

**Have the authors made all data and (if applicable) computational code underlying the findings in their manuscript fully available?**

The PLOS Data policy requires authors to make all data and code underlying the findings described in their manuscript fully available without restriction, with rare exception (please refer to the Data Availability Statement in the manuscript PDF file). The data and code should be provided as part of the manuscript or its supporting information, or deposited to a public repository. For example, in addition to summary statistics, the data points behind means, medians and variance measures should be available. If there are restrictions on publicly sharing data or code —e.g. participant privacy or use of data from a third party—those must be specified.

Reviewer #1: Yes

Reviewer #2: Yes

**********

PLOS authors have the option to publish the peer review history of their article (what does this mean?). If published, this will include your full peer review and any attached files.

If you choose “no”, your identity will remain anonymous but your review may still be made public.

**Do you want your identity to be public for this peer review?**  For information about this choice, including consent withdrawal, please see our Privacy Policy.

Reviewer #1: No

Reviewer #2: No

[NOTE: If reviewer comments were submitted as an attachment file, they will be attached to this email and accessible via the submission site. Please log into your account, locate the manuscript record, and check for the action link "View Attachments". If this link does not appear, there are no attachment files.]

**Figure resubmission:**

**Reproducibility:**

?>

Attachment Submitted filename: PLOS-CB-vaccine-nationalism-Dec-2025-authors.pdf

# Author comment: Author response to Decision Letter 1

**DOI:** 10.1371/journal.pcbi.1014466.r002

6 Apr 2026

Attachment Submitted filename: vaccine_nationalism_response_to_reviewers.pdf

# Peer review: Decision Letter 1

**DOI:** 10.1371/journal.pcbi.1014466.r003

**Authors:** Roger Dimitri Kouyos, Michael J Plank

29 May 2026

PCOMPBIOL-D-25-02016R1

Economic factors promoting vaccine nationalism in the face of viral evolution

PLOS Computational Biology

Dear Dr. Freedman,

Thank you for submitting your manuscript to PLOS Computational Biology. After careful consideration, we feel that it has merit but does not fully meet PLOS Computational Biology's publication criteria as it currently stands. Therefore, we invite you to submit a revised version of the manuscript that addresses the points raised during the review process.

Please submit your revised manuscript by Jul 29 2026 11:59PM. If you will need more time than this to complete your revisions, please reply to this message or contact the journal office at ploscompbiol@plos.org. When you're ready to submit your revision, log on to https://www.editorialmanager.com/pcompbiol/ and select the 'Submissions Needing Revision' folder to locate your manuscript file.

Please include the following items when submitting your revised manuscript:

* A letter that responds to each point raised by the editor and reviewer(s). You should upload this letter as a separate file labeled 'Response to Reviewers'. This file does not need to include responses to formatting updates and technical items listed in the 'Journal Requirements' section below.

* A marked-up copy of your manuscript that highlights changes made to the original version. You should upload this as a separate file labeled 'Revised Manuscript with Track Changes'.

* An unmarked version of your revised paper without tracked changes. You should upload this as a separate file labeled 'Manuscript'.

If you would like to make changes to your financial disclosure, competing interests statement, or data availability statement, please make these updates within the submission form at the time of resubmission. Guidelines for resubmitting your figure files are available below the reviewer comments at the end of this letter.

As the corresponding author, your ORCID iD is verified in the submission system and will appear in the published article. PLOS supports the use of ORCID, and we encourage all coauthors to register for an ORCID iD and use it as well. Please encourage your coauthors to verify their ORCID iD within the submission system before final acceptance, as unverified ORCID iDs will not appear in the published article. Only the individual author can complete the verification step; PLOS staff cannot verify ORCID iDs on behalf of authors.

We look forward to receiving your revised manuscript.

Kind regards,

Michael J Plank

Academic Editor

PLOS Computational Biology

Roger Kouyos

Section Editor

PLOS Computational Biology

**Additional Editor Comments:**

AE comments:

The ms has been re-reviewed by Reviewer #2 who still mounts strong criticisms of the study (Reviewer #1 was in favour of publication). Having read Reviewer #2’s original and latest sets of comments and re-read the ms, I am of the view that the ms can meet PLoS CB’s publication criteria with a further minor revision. In my view, the authors have provided a sound justification for the use of the two-country model with the full epidemiological dynamics, rather than the simpler model originally suggested by Reviewer #2. I am also satisfied that there is sufficient methodological interest and novelty in the relationship between the epidemiological dynamics and the economic outcomes to justify publication.

The Reviewer is still of the view that some of the economic modelling assumptions, particularly around the modelling of altruism, are naïve. The results about the effects of prosocialty are certainly unsurprising and less interesting than the effect of epidemiological and evolutionary parameters such as R0 and the mutation rate.

On balance, I am recommending a minor revision decision. The authors should shorten or de-emphasise the section about the effects of pro-sociality on model results. And they should add some specific caveats to section 3 acknowledging that pro-sociality is modelled in a narrow and highly simplified way, and the possibility that more efficient outcomes could be obtained by cash transfers rather than vaccine donations. Provided the authors can make these revisions in a satisfactory way the ms will not need to be sent for another round of peer review.

**Reviewers' comments:**

Reviewer's Responses to Questions

**Comments to the Authors:**

**Please note here if the review is uploaded as an attachment.**

Reviewer #2: None.

**********

**Have the authors made all data and (if applicable) computational code underlying the findings in their manuscript fully available?**

The PLOS Data policy requires authors to make all data and code underlying the findings described in their manuscript fully available without restriction, with rare exception (please refer to the Data Availability Statement in the manuscript PDF file). The data and code should be provided as part of the manuscript or its supporting information, or deposited to a public repository. For example, in addition to summary statistics, the data points behind means, medians and variance measures should be available. If there are restrictions on publicly sharing data or code —e.g. participant privacy or use of data from a third party—those must be specified.

Reviewer #2: Yes

**********

PLOS authors have the option to publish the peer review history of their article (what does this mean?). If published, this will include your full peer review and any attached files.

If you choose “no”, your identity will remain anonymous but your review may still be made public.

**Do you want your identity to be public for this peer review?**  For information about this choice, including consent withdrawal, please see our Privacy Policy.

Reviewer #2: No

[NOTE: If reviewer comments were submitted as an attachment file, they will be attached to this email and accessible via the submission site. Please log into your account, locate the manuscript record, and check for the action link "View Attachments". If this link does not appear, there are no attachment files.]

**Figure resubmission:**

**Reproducibility:**

To enhance the reproducibility of your results, we recommend that authors of applicable studies deposit laboratory protocols in protocols.io, where a protocol can be assigned its own identifier (DOI) such that it can be cited independently in the future. Additionally, PLOS ONE offers an option to publish peer-reviewed clinical study protocols. Read more information on sharing protocols at https://plos.org/protocols?utm_medium=editorial-email&utm_source=authorletters&utm_campaign=protocols

# Author comment: Author response to Decision Letter 2

**DOI:** 10.1371/journal.pcbi.1014466.r004

16 Jun 2026

Attachment Submitted filename: vaccine_nationalism_response_to_reviewers_round_2.pdf

# Editor report: Decision Letter 2

**DOI:** 10.1371/journal.pcbi.1014466.r005

**Authors:** Roger Dimitri Kouyos, Michael J Plank

18 Jun 2026

Dear Freedman,

We are pleased to inform you that your manuscript 'Economic factors promoting vaccine nationalism in the face of viral evolution' has been provisionally accepted for publication in PLOS Computational Biology.

Before your manuscript can be formally accepted you will need to complete some formatting changes, which you will receive in a follow up email. A member of our team will be in touch with a set of requests.

Please note that your manuscript will not be scheduled for publication until you have made the required changes, so a swift response is appreciated.

IMPORTANT: The editorial review process is now complete. PLOS will only permit corrections to spelling, formatting or significant scientific errors from this point onwards. Requests for major changes, or any which affect the scientific understanding of your work, will cause delays to the publication date of your manuscript.

Should you, your institution's press office or the journal office choose to press release your paper, you will automatically be opted out of early publication. We ask that you notify us now if you or your institution is planning to press release the article. All press must be co-ordinated with PLOS.

Thank you again for supporting Open Access publishing; we are looking forward to publishing your work in PLOS Computational Biology.

Best regards,

Michael J Plank

Academic Editor

PLOS Computational Biology

Roger Kouyos

Section Editor

PLOS Computational Biology

***********************************************************

AE comments: the authors have made the revisions requested and I am pleased to recommend the paper for publication.

# Editor report: Acceptance letter

**DOI:** 10.1371/journal.pcbi.1014466.r006

**Authors:** Roger Dimitri Kouyos, Michael J Plank

PCOMPBIOL-D-25-02016R2

Economic factors promoting vaccine nationalism in the face of viral evolution

Dear Dr Freedman,

I am pleased to inform you that your manuscript has been formally accepted for publication in PLOS Computational Biology. Your manuscript is now with our production department and you will be notified of the publication date in due course.

The corresponding author will soon be receiving a typeset proof for review, to ensure errors have not been introduced during production. Please review the PDF proof of your manuscript carefully, as this is the last chance to correct any errors. Please note that major changes, or those which affect the scientific understanding of the work, will likely cause delays to the publication date of your manuscript.

Soon after your final files are uploaded, unless you have opted out, the early version of your manuscript will be published online. The date of the early version will be your article's publication date. The final article will be published to the same URL, and all versions of the paper will be accessible to readers.

For Research, Software, and Methods articles, you will receive an invoice from PLOS for your publication fee after your manuscript has reached the completed accept phase. If you receive an email requesting payment before acceptance or for any other service, this may be a phishing scheme. Learn how to identify phishing emails and protect your accounts at https://explore.plos.org/phishing.

Thank you again for supporting PLOS Computational Biology and open-access publishing. We are looking forward to publishing your work!

With kind regards,

Anitha Samidurai

PLOS Computational Biology | Carlyle House, Carlyle Road, Cambridge CB4 3DN | United Kingdom ploscompbiol@plos.org | Phone +44 (0) 1223-442824 | ploscompbiol.org | @PLOSCompBiol
